# Adaptation of codon and amino acid use for translational functions in highly expressed cricket genes

**DOI:** 10.1186/s12864-021-07411-w

**Published:** 2021-04-06

**Authors:** Carrie A. Whittle, Arpita Kulkarni, Nina Chung, Cassandra G. Extavour

**Affiliations:** 1 Department of Organismic and Evolutionary Biology, Harvard University, 16 Divinity Avenue, Cambridge, MA 02138 USA; 2 Department of Molecular and Cellular Biology, Harvard University, 16 Divinity Avenue, Cambridge, 02138 MA USA

**Keywords:** Codon, Amino acid, Tissue-type, Translational selection, Regulation, tRNAs

## Abstract

**Background:**

For multicellular organisms, much remains unknown about the dynamics of synonymous codon and amino acid use in highly expressed genes, including whether their use varies with expression in different tissue types and sexes. Moreover, specific codons and amino acids may have translational functions in highly transcribed genes, that largely depend on their relationships to tRNA gene copies in the genome. However, these relationships and putative functions are poorly understood, particularly in multicellular systems.

**Results:**

Here, we studied codon and amino acid use in highly expressed genes from reproductive and nervous system tissues (male and female gonad, somatic reproductive system, brain and ventral nerve cord, and male accessory glands) in the cricket *Gryllus bimaculatus*. We report an optimal codon, defined as the codon preferentially used in highly expressed genes, for each of the 18 amino acids with synonymous codons in this organism. The optimal codons were mostly shared among tissue types and both sexes. However, the frequency of optimal codons was highest in gonadal genes. Concordant with translational selection, a majority of the optimal codons had abundant matching tRNA gene copies in the genome, but sometimes obligately required wobble tRNAs. We suggest the latter may comprise a mechanism for slowing translation of abundant transcripts, particularly for cell-cycle genes. Non-optimal codons, defined as those least commonly used in highly transcribed genes, intriguingly often had abundant tRNAs, and had elevated use in a subset of genes with specialized functions (gametic and apoptosis genes), suggesting their use promotes the translational upregulation of particular mRNAs. In terms of amino acids, we found evidence suggesting that amino acid frequency, tRNA gene copy number, and amino acid biosynthetic costs (size/complexity) had all interdependently evolved in this insect model, potentially for translational optimization.

**Conclusions:**

Collectively, the results suggest a model whereby codon use in highly expressed genes, including optimal, wobble, and non-optimal codons, and their tRNA abundances, as well as amino acid use, have been influenced by adaptation for various functional roles in translation within this cricket. The effects of expression in different tissue types and the two sexes are discussed.

**Supplementary Information:**

The online version contains supplementary material available at 10.1186/s12864-021-07411-w.

## Background

Synonymous codons in protein-coding genes are not used randomly [1]. The preferential use of synonymous codons per amino acid in highly transcribed genes, often called optimal codons, has been observed in diverse organisms including bacteria, fungi, plants and animals [2–18], including insects such as flies, mosquitoes, beetles and crickets [10, 11, 19–23]. When optimal codons co-occur with a high count of iso-accepting tRNA gene copies in the genome, which reflects an organism’s tRNA abundance [3–5, 12, 24–27], it suggests a history of selection favoring translational optimization [1, 3, 5, 12, 21, 23, 27–31]. In multicellular organisms, unlike unicellular systems, genes can be expressed at different levels among tissue types and between the two sexes [20, 32–35]. Thus, in these organisms, codon use may be more complex, given that it is plausible that optimal codons may depend on the tissue type or sex in which a gene is expressed [11, 20, 28, 36, 37], and codon use could feasibly adapt to local tissue-dependent tRNA populations [36, 38, 39]. However, only minimal data are currently available about whether and how codon use varies with high expression in different tissue types and between the two sexes in multicellular organisms.

The limited data that are available suggest that codon use varies among genes transcribed in different tissues. We recently found, for example, that some optimal codons of highly transcribed genes differed among males and females for the testis, ovaries, gonadectomized-males and gonadectomized females, which may suggest adaptation of codon use to local tRNA populations in the beetle *Tribolium castaneum* [20]. In addition, a study in *Drosophila melanogaster* showed that certain codons were preferentially used in the testis (CAG (Gln), AAG (Lys), CCC (Pro), and CGU (Arg)) as compared to other tissues such as the midgut, ovaries, and salivary glands, a result that was taken as support for the existence of tissue-specific tRNA populations [38] (see also an analysis of codon bias by [37]). Similar patterns of tissue-related use of specific codons have been inferred in humans [39, 40] and the plants *Arabidopsis thaliana* and *Oryza sativa* [36, 41]. Given the limited scope of organisms studied to date, however, further research is needed to determine whether the codon use varies among tissues across a broader scale of systems. Tissues that are of particular importance for research include the gonads, which are key to reproductive success, and the brain, wherein the transcribed genes are apt to regulate male and female sexual behaviors [42–44]. Translational optimization of highly transcribed genes in these tissues may be particularly significant for an organism’s fitness.

While much of the focus on codon use in an organism’s highly expressed genes to date has centered on optimal codons [3, 5, 7, 12, 15–17, 20, 21, 23, 28–31], and whether they have abundant matching tRNAs that may improve translation [3, 12, 21, 23, 27–30], growing evidence suggests that other, less well studied, types of codon statuses could also play important translational roles [45–47]. In particular, even for codons that are not optimal per se, the supply-demand relationship between codons and tRNA abundances may regulate translation rates, possibly affecting protein functionality and abundance [45, 48–50]. For example, in vivo experimental research has shown that genes using codons requiring wobble tRNAs, which imprecisely match a codon at the third nucleotide site, exhibit slowed movement of ribosomes along mRNAs [45, 51, 52]. Similarly, non-optimal codons, defined as those codons that are least commonly used in highly transcribed genes (or sometimes defined as “rare” codons), particularly those non-optimal codons with few or no tRNAs in the cellular tRNA pool [20], may decelerate translation and thereby prevent ribosomal jamming [26] and also allow proper co-translational protein folding [47, 53–56]. In this regard, wobble codons, and non-optimal codons with few matching tRNA gene copies in the genome, may have significant translational roles, including roles in slowing translation.

In contrast to non-optimal codons that have few tRNAs, some evidence has emerged suggesting non-optimal codons may sometimes have abundant tRNAs, a relationship that may act to improve translation of specific gene mRNAs [20, 48]. For instance, in yeast (*Saccharomyces cerevisiae*), rare genomic codons exhibit enhanced use in stress genes, and tRNAs matching these codons have been found to be upregulated in response to stressful conditions, allowing improvement of their translation levels without any change in transcription rates [48]. In the red flour beetle, we recently reported that some non-optimal codons have abundant matching tRNA genes in the genome [20], and these codons are concentrated in a subset of highly transcribed genes with specific, non-random, biological functions (e.g., olfactory or stress roles), which may together allow preferential translation of mRNAs of those particular genes [20]. Accordingly, given these findings, further studies of codon use patterns in highly expressed genes of multicellular organisms should expand beyond the focus on optimal codons per se [2, 3, 7–9, 12, 15, 17, 23], and explore the use and possible translational functions of non-optimal codons, distinguishing between those that have few and plentiful tRNAs, as well as the use of wobble codons [20].

While the investigation of amino acid use in highly transcribed genes remains uncommon in multicellular organisms, the available sporadic studies suggest an association between amino acid use and gene expression level [10, 23, 57]. In insects, for example, an assessment of the biosynthetic costs of amino acid synthesis (size/complexity score for each of 20 amino acids as quantified by Dufton [58]) has shown that those amino acids with low costs tend to be more commonly used in genes with high transcription levels in the beetle *T. castaneum* [23]. Further, genome-wide studies in other arthropod models such as spiders (*Parasteatoda tepidariorum*) [57], and the study of available transcriptomes from milkweed bugs (*Oncopeltus fasciatus*), an amphipod crustacean (*Parhyale hawaiensis*) and crickets (*Gryllus bimaculatus*, using a single ovary/embryo dataset in this system) [10], were suggestive of the hypothesis that evolution may have typically favored a balance between minimizing the amino acid costs for production of abundant proteins with the need for certain (moderate cost) amino acids to ensure proper protein function (protein stability and/or functionality) [10]. Moreover, it has been found that amino acid use is correlated to their tRNA gene copy numbers in beetles [23], and in some other eukaryotes [24], a relationship that may be stronger in highly transcribed genes [24]. Thus, these various patterns raise the possibility of adaptation of amino acid use for translational optimization in multicellular organisms [23, 24, 57]. At present, further data is needed on amino acid use in highly expressed genes in multicellular systems, that include consideration of tRNA gene number, biosynthetic costs, and expression in different tissue types.

An emerging model system that provides opportunities for further deciphering the relationships between gene expression and codon and amino acid use is the two-spotted cricket *Gryllus bimaculatus*. Within insects, *Gryllus* is a hemimetabolous genus (Order Orthoptera) and has highly diverged from the widely studied model insect genus *Drosophila* (Order Diptera) [59, 60]. *G. bimaculatus* comprises a model for investigations in genetics [61, 62], germ line formation and development [63–65] and for molecular evolutionary biology [10, 66]. In the present study, we rigorously assess codon and amino acid use in highly transcribed genes of *G. bimaculatus* using its recently available annotated genome [67] and large-scale RNA-seq data from tissues of the male and female reproductive and nervous systems [66]. From our analyses, we provide evidence suggesting that optimal codons, those preferentially used in highly expressed genes, occur in this organism, are influenced by selection pressures, and are nearly identical across tissues. Based on analyses of codon and tRNA gene copy relationships, we find that a majority of optimal codons have abundant tRNAs, which is consistent with translational optimization in this species. However, some optimal codons obligately require the use of wobble tRNAs, which may act to slow translation, including for cell-cycle genes. Moreover, non-optimal codons, those codons rarely used in highly expressed genes, rather than usually having few tRNAs, often have abundant tRNAs, and thus may provide a system to upregulate the translation of specific mRNAs (for example, apoptosis gonadal genes), as has been proposed in yeast and beetles [20, 48]. Finally, with respect to amino acids, we find evidence to suggest that amino acid frequency, tRNA gene copy number, and amino acid biosynthetic costs have all interdependently evolved in this taxon, possibly for translational optimization.

## Results

For our study, codon and amino acid use in *G. bimaculatus* was assessed using genes from its recently available annotated genome [67]. We included all 15,539 *G. bimaculatus* protein-coding genes (CDS, longest CDS per gene) that had a start codon and were >150 bp. Gene expression (FPKM) was assessed using RNA-seq data from four adult male and female tissue types, the gonad (testis for males, ovaries for females), somatic reproductive system (for males this includes the pooled vasa deferentia, seminal vesicle and ejaculatory duct, and for females includes the spermathecae, common oviduct, and bursa), brain and ventral nerve cord (Additional file 1: Table S1 [66]). The male accessory glands were included for study, but were separated from the other male reproductive system elements to prevent overwhelming, or skewing, the types of transcripts detected in the former tissues [66]. To identify and study the optimal and non-optimal codons in *G. bimaculatus*, we compared codon use in highly versus lowly expressed genes [2, 7, 9, 10, 15, 19, 20, 22, 68]. For each CDS, the relative synonymous codon usage (RSCU) was determined for all codons for each amino acid with synonymous codons [25], which was used to assess the ∆RSCU = RSCU_Mean Highly Expressed CDS_-RSCU_Mean Lowly Expressed CDS_. The primary optimal codon was defined as the codon with the largest positive and statistically significant ∆RSCU value per amino acid [2, 7, 9, 10, 15, 19, 20]. The primary non-optimal codon was defined as the codon with the largest negative and statistically significant ∆RSCU value per amino acid [20].

In the following sections, we first thoroughly describe the optimal codons identified in this cricket species at the organism-wide level, and within each of the individual tissue types, and consider the relative role of selection versus mutation in shaping the optimal codons. Subsequently, we evaluate the relationships between optimal codons and non-optimal codons and their matching tRNA gene counts in the genome to ascertain plausible functional roles. We then consider the amino acid use and tRNA relationships in highly expressed genes of this taxon.

### Optimal codons are shared across the nine distinct tissues in *G. bimaculatus*

The organism-wide optimal codons were identified for *G. bimaculatus* using ΔRSCU for genes with the top 5% average expression levels across all nine studied tissues (cutoff was 556.2 FPKM) versus the 5% of genes with the lowest average expression levels (among all 15,539 genes under study) and are shown in Table 1. Based on ΔRSCU we report a primary optimal codon for all of the 18 amino acids with synonymous codons, each of which ended at the third position in an A (A3) or T (T3) nucleotide (Table 1). As shown in Table 2, the 777 genes in the top 5% average expression category (organism-wide analysis) were enriched for ribosomal protein genes and had mitochondrial and protein folding functions. We found that 14 of the 17 primary optimal codons (one per amino acid) that were previously identified using a partial transcriptome from one pooled tissue sample (embryos/ovaries [10]) were identical to those observed here, marking a strong concordance between studies and datasets (the differences herein were CAA for Gln, TTA for Leu, and AGA for Arg as optimal codons, and the presence of an optimal codon AAA for Lys, which had no optimal codon using previous embryonic/ovary data [10]). Thus, the present analysis using large-scale RNA-seq from nine divergent tissues (Additional file 1: Table S1) and using a complete annotated genome [67] support a strong preference for AT3 codons in the most highly transcribed genes of this cricket.

**Table 1:** The organism-wide ΔRSCU values determined using genes with the top 5% expression level (when averaged across all nine tissues) and lowest 5% expression level (**P < 0.001), the predicted tRNA numbers, and codon statuses.

Amino acid	Codon (DNA)	Standard anticodon	ΔRSCU	P^a^	No. tRNAs	Optimal and non-optimal status	Wobble anticodon (optimal)^b^
Ala	**GCT**	**AGC**	**+ 0.871**	**	35	Opt-codon_↑tRNAs_	
Ala	GCC	GGC	−0.344	**	0	–	
Ala	GCA	UGC	+ 0.518	**	18	–	
Ala	GCG	CGC	−1.039	**	22	Nonopt-codon_↑tRNAs_	
Arg	CGT	ACG	+ 0.463	**	40	–	
Arg	CGC	GCG	−1.053	**	0	Nonopt-codon_↓tRNAs_	
Arg	CGA	UCG	+ 0.185	**	39	–	
Arg	CGG	CCG	−0.548	**	2	–	
Arg	**AGA**	**UCU**	**+ 0.881**	**	18	Opt-codon_↑tRNAs_	
Arg	AGG	CCU	+ 0.047		26	–	
Asn	**AAT**	**AUU**	**+ 0.416**	**	0	Opt-codon_wobble_	GUU
Asn	AAC	GUU	−0.244	**	37	Nonopt-codon_↑tRNAs_	
Asp	**GAT**	**AUC**	**+ 0.520**	**	0	Opt-codon_wobble_	GUC
Asp	GAC	GUC	−0.482	**	31	Nonopt-codon_↑tRNAs_	
Cys	**TGT**	**ACA**	**+ 0.368**	**	0	Opt-codon_wobble_	GCA
Cys	TGC	GCA	−0.365	**	38	Nonopt-codon_↑tRNAs_	
Gln	**CAA**	**UUG**	**+ 0.254**	**	39	Opt-codon_↑tRNAs_	
Gln	CAG	CUG	−0.218	**	37	Nonopt-codon_↑tRNAs_	
Glu	**GAA**	UUC	**+ 0.496**	**	31	Opt-codon_↑tRNAs_	
Glu	GAG	CUC	−0.480	**	18	Nonopt-codon_↑tRNAs_	
Gly	**GGT**	**ACC**	**+ 0.610**	**	0	Opt-codon_wobble_	GCC
Gly	GGC	GCC	−0.709	**	41	Nonopt-codon_↑tRNAs_	
Gly	GGA	UCC	+ 0.483	**	19	–	
Gly	GGG	CCC	−0.383	**	11	–	
His	**CAT**	**AUG**	**+ 0.511**	**	0	Opt-codon_wobble_	GUG
His	CAC	GUG	−0.452	**	37	Nonopt-codon_↑tRNAs_	
Ile	**ATT**	**AAU**	**+ 0.603**	**	22	Opt-codon_↑tRNAs_	
Ile	ATC	GAU	−0.452	**	0	Nonopt-codon_↓tRNAs_	
Ile	ATA	UAU	+ 0.045		19	–	
Leu	**TTA**	**UAA**	**+ 0.537**	**	28	Opt-codon_↑tRNAs_	
Leu	TTG	CAA	+ 0.383	**	16	–	
Leu	CTT	AAG	+ 0.409	**	39	–	
Leu	CTC	GAG	−0.629	**	0	–	
Leu	CTA	UAG	+ 0.007		28	–	
Leu	CTG	CAG	−0.692	**	30	Nonopt-codon_↑tRNAs_	
Lys	**AAA**	**UUU**	**+ 0.263**	**	20	Opt-codon_↑tRNAs_	
Lys	AAG	CUU	−0.160	**	50	Nonopt-codon_↑tRNAs_	
Phe	**TTT**	**AAA**	**+ 0.407**	**	0	Opt-codon_wobble_	GAA
Phe	TTC	GAA	−0.265	**	48	Nonopt-codon_↑tRNAs_	
Pro	**CCT**	**AGG**	**+ 0.749**	**	36	Opt-codon_↑tRNAs_	
Pro	CCC	GGG	−0.359	**	0	–	
Pro	CCA	UGG	+ 0.483	**	31	–	
Pro	CCG	CGG	−0.843	**	36	Nonopt-codon_↑tRNAs_	
Ser	**TCT**	**AGA**	**+ 0.731**	**	36	Opt-codon_↑tRNAs_	
Ser	TCC	GGA	−0.208	**	0	–	
Ser	TCA	UGA	+ 0.493	**	21	–	
Ser	TCG	CGA	−0.723	**	15	Nonopt-codon_↑tRNAs_	
Ser	AGT	ACU	+ 0.325	**	0	–	
Ser	AGC	GCU	−0.619	**	60	–	
Thr	**ACT**	**AGU**	**+ 0.644**	**	35	Opt-codon_↑tRNAs_	
Thr	ACC	GGU	−0.223	**	0	–	
Thr	ACA	UGU	+ 0.493	**	37	–	
Thr	ACG	CGU	−0.873	**	31	Nonopt-codon_↑tRNAs_	
Tyr	**TAT**	**AUA**	**+ 0.430**	**	0	Opt-codon_wobble_	GUA
Tyr	TAC	GUA	−0.186	**	43	Nonopt-codon_↑tRNAs_	
Val	**GTT**	**AAC**	**+ 0.600**	**	26	Opt-codon_↑tRNAs_	
Val	GTC	GAC	−0.394	**	0	–	
Val	GTA	UAC	+ 0.314	**	30	–	
Val	GTG	CAC	−0.484	**	40	Nonopt-codon_↑tRNAs_	
**Amino acids with one codon**							
Met	ATG	CAU			43	–	
Trp	TGG	CCA			32	–	
**Total tRNAs**					1391		

The number of predicted tRNAs are shown [69]. The primary optimal codon per amino acid and its ΔRSCU value are in bold and underlined. The status of an optimal codon that has a relatively high number of tRNAs (≥18) and those with no tRNAs, and thus obligately requiring the use of wobble tRNAs, are shown, as well as the putative wobble anticodon. The status of primary non-optimal codons that have matching tRNA gene numbers substantially in excess of 0 (≥15) and those with few/no tRNAs are indicated. The status categories are further described in the main text. Codons not having primary optimal or non-optimal status are indicated by “--“.^a^, α = 0.05, all "**" contrasts had P < 0.001, including after Bonferonni correction. ^b^ Standard wobble codons provided; see also inosine modified anticodons for codons with no exact matching tRNAs [70, 71].

**Table 2:** Top predicted GO functional groups for organism-wide highly expressed genes (top 5% expression levels when averaged FPKM across all nine tissues). The top clusters with the greatest enrichment (abundance) scores are shown. *P*-values are derived from a modified Fisher’s test, where lower values indicate greater enrichment. Data is from DAVID software [72] using those *G. bimaculatus* genes with *D. melanogaster* orthologs (BLASTX e < 10^− 3^ [73]).

Enrichment Score: 18.88	P-value
Ribosomal protein	7.30X10^− 31^
Cytosolic ribosome	9.00 X10^− 11^
**Enrichment Score: 12.49**	
Mitochondrion	3.50 X10^−17^
**Enrichment Score: 8.39**	
Electron transport	1.90 X10^−10^
Respiratory chain	1.20X10^−9^
**Enrichment Score: 6.49**	
Protein folding	2.40 X10^−10^

Importantly, the expression datasets herein (Additional file 1: Table S1) allowed us to also conduct an assessment of whether the identity of optimal codons varied with tissue type or sex. As certain data suggest that codon use may be influenced by the tissue in which it is maximally transcribed [20, 36], we examined those genes that exhibited maximal expression (in the top 5%) within each tissue type, that were not in the top 5% for any of the other eight remaining tissue types [20, 36], which we refer to as Top5_One-tissue_ (N values as follows: female gonad (274), male gonad (270), female somatic reproductive system (67), male somatic reproductive system (104), female brain (24), male brain (22); female ventral nerve cord (32); male ventral nerve cord (33), and male accessory glands (162)). We emphasize that the Top5_One-tissue_ gene set for each tissue type is mutually exclusive of the top 5% expressed genes in any other tissue, but could be expressed in other tissues (outside the top 5%). We found remarkable consistency among tissues, with nearly all identified optimal codons (largest positive ΔRSCU and *P* < 0.05) ending in A3 and T3 in each tissue (Additional file 1: Table S2). For amino acids with two codons, the organism-wide optimal codon was consistently optimal across all nine tissues (Additional file 1: Table S2; with a possible exception for CAG for Gln in the male brain; however this had *P* > 0.1, and the N values and thus statistical power was lowest for the male brain; Additional file 1: Table S2). Nonetheless, there was some minor variation among the AT3-ending codons for amino acids with three or more synonymous codons. As an example, for the amino acid Thr, ACT was the optimal codon at the organism-wide level (Table 1) and for five tissues types (male somatic reproductive system, male brain, male ventral nerve cord, female ventral nerve cord, and male accessory glands), while the secondary organism-wide optimal codon ACA (secondary status is based on their magnitude of +ΔRSCU values) was the primary optimal codon in four other tissues (Additional file 1: Table S2). Thus, for some amino acids there is mild variation in primary and secondary status among tissues of the AT3 codons, which may reflect modest differences in the tRNA abundances among tissues [20, 38]. However, the overall patterns suggest there is remarkably high consistency in the identity of AT3 optimal codons across diverse tissues in this taxon (Additional file 1: Table S2).

While other studies of tissue-related optimal codons in multicellular organisms have been uncommon, the data available from fruit flies, thale cress (*Arabidopsis*), and our recent results from red flour beetles [20, 36, 38] have shown that optimal codons can vary among tissues, which suggests the existence of tissue-specific tRNA pools in those taxa [38]. The results here in *G. bimaculatus* thus differ from those in other organisms, and suggest its tRNA pools may vary only minimally with tissue or sex. Future studies using direct quantification of tRNA populations in various tissue types, which is a methodology under refinement and wherein the most effective approaches remain debated [48, 74], will help further affirm whether tRNA populations are largely similar among tissues and sex in this organism. Taken together, the results from this Top5_One-tissue_ analysis suggest that high transcription in even a single tissue type or sex is enough to give rise to the optimal codons in this species. We note nonetheless that while the identity of optimal codons (as AT3 ending codons), and thus potentially the relative tRNA abundances, are shared among genes expressed in different tissues, the degree of use of these codons (frequency of optimal codons (Fop) [28]) varied among tissue types (Top5_One-tissue_). Thus, the absolute levels of tRNAs may differ among tissues (see below section “*Fop varies with tissue type and sex*”).

#### Selective pressure is a factor shaping optimal codons

Given that the optimal codons were highly consistent across tissues, to further investigate the potential role of selection in shaping the optimal codons we hereafter focused on the organism-wide optimal codons in Table 1 (which used averaged expression across all nine tissues to define optimal codons). While the elevated use of the specific types of codons in highly expressed genes in Table 1 in itself provides evidence suggesting a history of selection favoring the use of optimized codons in *G. bimaculatus* [2, 7, 9, 10, 19, 20, 22, 68], the putative role of selection can be further evaluated by studying the AT (or GC) content of introns (AT-I), which are thought to largely reflect background neutral pressures (mutational bias and biased gene conversion (BGC)) on genes, and thus on AT3 [20, 22, 75–79]. The *G. bimaculatus* genome contains repetitive A and T rich non-coding DNA [67], including in the introns. The AT-I content across all genes in this taxon had a median of 0.637, indicating a substantial background compositional nucleotide bias, and differing from the whole gene CDS (median AT for CDS across all sites = 0.525, AT3 = 0.546). Nonetheless, with this recognition, in order to decipher whether any additional insights might be gained from the introns in *G. bimaculatus* we extracted the introns from genes across the entire genome and found that 90.5% (*N* = 14,071) of the 15,539 annotated genes had introns suitable for study (≥50 bp after trimming). Introns (longest per gene) were nearly two- fold shorter for the most highly (top 5% organism-wide) than lowly (lowest 5%) expressed genes (1.91 fold longer in low than high expressed genes, MWU-test *P* = 8.9X10^− 16^). We speculate that the shorter introns under high expression may comprise a mechanism to minimize transcriptional costs of abundantly produced transcripts in this cricket, as has been suggested in some other species including humans and nematodes [80], and may indicate a history of some non-neutral evolutionary pressures on the length of introns.

To further distinguish the role of mutation from selection in shaping AT3 in this cricket, we evaluated the relationship between gene expression (FPKM) and AT-I and AT3. We found that AT-I was positively correlated to gene expression level (using averaged expression across all tissues per gene), with Spearman’s R = 0.354, *P* < 2X10^− 7^ across the 14,071 annotated genes with introns. Thus, assuming intron nucleotide content is largely due to neutral (non-adaptive) processes, this may suggest a degree of expression-linked mutational bias [81, 82] in this organism favoring AT mutations in introns as transcription increases (or conversely, elevated GC mutations at low expression levels, see below in this section). However, this correlation was weaker than that observed between AT3 of protein-coding genes and expression across these same genes (R = 0.534, P < 2X10^− 7^), thus suggesting that selection is also a significant force that shapes AT3 in the genome [8], a factor that may be particularly apt to influence AT3 in the most highly expressed genes.

**Fig. 1:**
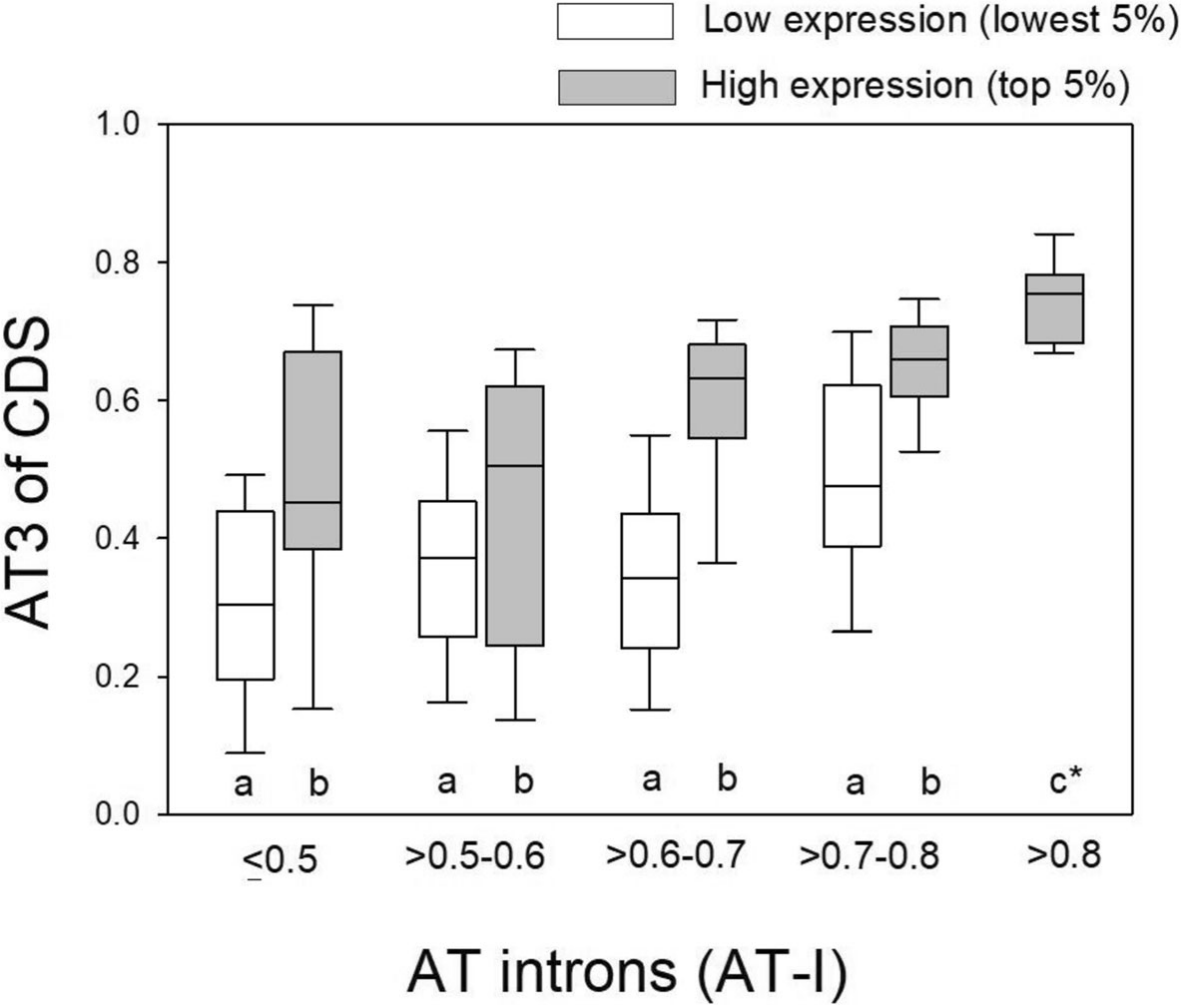
Box plots of the AT3 of codons of lowly and highly expressed genes within narrow bins of AT-I, and thus presumably having similar background mutational pressures. Genes were binned into categories with similar AT-I content to ascertain differences in AT3 with respect to expression. Different letters in each pair of bars indicates *P* < 0.05 using MWU-tests. No statistically significant differences in AT-I were observed between highly and lowly expressed genes for any bins (MWU-test P > 0.30; with the exception of a minor AT-I difference in medians of 0.019 for category 3 (0.6–0.7)). *AT3 for this bar is statistically significant from all other bars. Only one gene had AT-I > 0.8 for lowly expressed genes and thus the bar for this category was excluded.

For additional rigor in verifying the role of selection in favoring AT3 codons, as compared to mutation, in highly expressed genes (Table 1), genes from the top 5% and lowest 5% gene expression categories were placed into one of five narrow bins based on their AT-I content, specifically ≤0.5, > 0.5–0.6, > 0.6–0.7, > 0.7–0.8, and > 0.8. As shown in Fig. 1, for each AT-I bin, we found that AT3 of the top 5% expressed genes was statistically significantly higher than that of lowly expressed genes (MWU-tests P between 0.01 and < 0.001). No differences in AT-I between highly and lowly expressed genes were observed per bin (MWU-test *P* > 0.30 in all bins, with one exception of a minimal median AT-I difference of 0.019 for category 3 (*P* < 0.05), Fig. 1). Thus, this explicitly demonstrates that within genes that have a similar background intron nucleotide composition (that is, genes contained in one narrow bin of AT-I values), AT3 codons exhibit significantly greater use in highly transcribed than in lowly transcribed genes. This pattern further supports the interpretation that selection substantially shapes optimal codon use in the highly expressed genes of *G. bimaculatus*.

As an additional consideration, we also considered whether the low AT3 content of lowly expressed genes (as indicated by ∆RSCU in Table 1, and in Fig. 1) could be related to biased gene conversion, which acts to enhance GC content [79, 83]. BGC is thought to arise from recombination during meiosis, whereby DNA repair may favor AT to GC conversions, which can elevate GC content of affected genes, and influence both coding and non-coding DNA regions [84–86]. BGC has been only minimally considered or excluded in studies of translational selection for optimal codons [2, 7, 9, 10, 15, 17, 19, 20, 22, 68], even though some evidence suggests it may influence codon patterns in certain organisms, particularly mammals [83, 85, 86]. Our interpretation of the collective data is that even if BGC occurs in this cricket species, it is not apt to explain the identified optimal codons in its highly expressed genes in Table 1. Specifically, in Fig. 1, elevated AT3 content of highly than lowly expressed genes was observed for each relative to lowly intron AT-I bin (where introns should largely reflect background BGC and mutational pressures [79, 86, 87], see also [88]). In addition, the relationships between codon use and tRNAs in Table 1 suggest translational selection (for details see below section “*Functional Roles of Optimal and Non-Optimal Codons Inferred by their Relationships to tRNA Gene Copies*”). Further, for each tissue type using genes with Top5_One-tissue_ status, whereby each highly expressed gene set per tissue was mutually exclusive of the gene sets from the eight other tissues, we found the same tendency for AT3 optimal codons (Additional file 1: Table S2), thus suggesting the pattern is robust to tissue type, including high expression in the testis and ovary (meiotic tissues where recombination occurs) and the various somatic tissues (see further consideration with respect to patterns observed in meiotic tissues in humans [83]; Additional file 1: Text file S1; and for a summary of the roles of selection see Discussion). Thus, we infer that while BGC may occur in this species and in turn influence background nucleotide composition and codon use in some genes, the evidence in Table 1, Fig. 1, and Additional file 1: Text file S1 suggest that within its most highly expressed genes, are the focus herein, selection has contributed to the use of AT3 codons.

It is worth noting that factors in addition to mutation or BGC may specifically influence the introns in this organism. For instance, we observed that AT3 trended lower than AT-I, particularly for the lowly expressed genes (comparison of AT-I on X-axis versus AT3 on Y-axis, Fig. 1). It may be speculated that AT-rich zones, possibly enriched in introns due to AT-rich transposons preferentially localizing to the introns (and not in CDS) [84, 86, 88], may have acted to enhance AT-I to a level beyond that resulting solely from background mutational AT-biases or BGC (or lack thereof) pressures. Further studies focused on the introns would be needed to further evaluate this possibility.

#### Fop varies with tissue type and sex

**Fig. 2:**
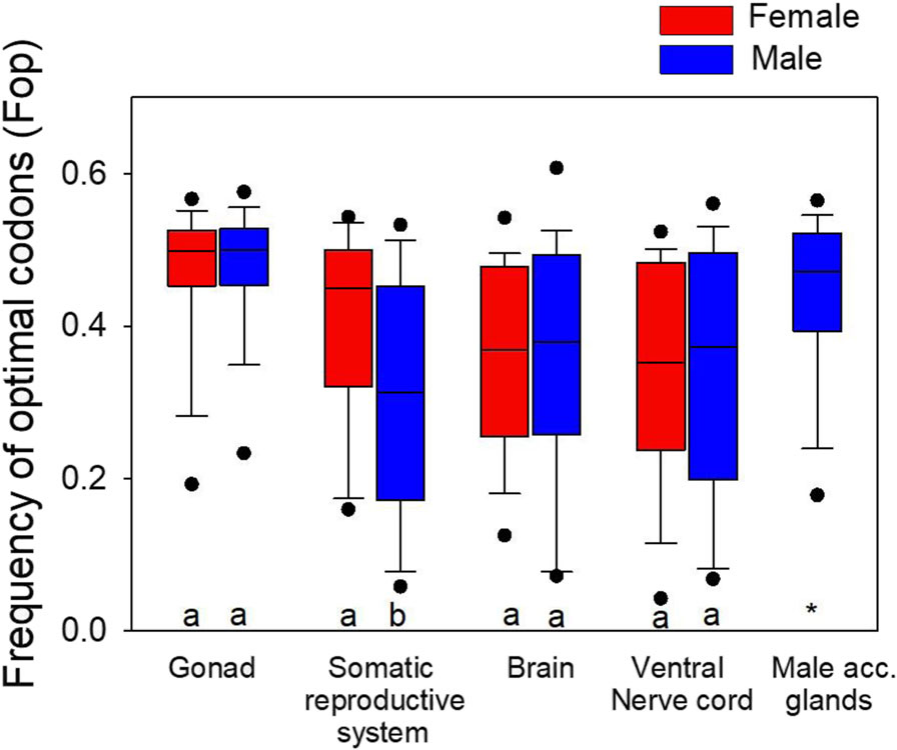
The frequency of optimal codons (Fop) for genes with expression in the top 5% in one tissue type and not in any other tissues (Top5_One-tissue_) for *G. bimaculatus*. Different letters within each pair of bars indicates a statistically significant difference (MWU-test P < 0.05). Note that the gonad (male and female) genes had higher Fop values than all other categories (MWU-tests P < 0.05). ^*^Indicates a difference of male accessory (acc.) gland genes from all other bars

While the identities of optimal codons identified herein were largely shared among tissues (Additional file 1: Table S2), the frequency of use of these codons (Fop) varied markedly with tissue type and sex in *G. bimaculatus*. In particular, Fop was markedly higher in Top5_One-tissue_ genes from the testes and ovaries and the male accessory glands, than in all other six tissue types (paired MWU-tests all have *P* < 0.05, Fig. 2). Thus, this suggests that genes linked to these fundamental sexual structures and functions are prone to elevated optimal codon use that could, in principle, be due to their essential roles in reproduction and fitness, and cost-efficient translation may be particularly beneficial in the contained haploid meiotic cells [20]. Moreover, we found that the Top5_One-tissue_ genes from the female somatic reproductive system had markedly higher Fop than their male counterparts (MWU-test *P* = 6.6X10^− 5^, Fig. 2). We speculate that this may reflect the essential and fitness-related roles of genes involved in the insect female structures since they transport and house the male sex cells and seminal fluids after mating [89, 90], possibly making translational optimization more consequential to reproductive success for the female than male genes. In contrast, no differences in Fop were observed with respect to sex for the brain or ventral nerve cord, and the relatively low Fop values for these tissues suggest weakened selective pressure on codon use of genes as compared to the gonads and to the male accessory glands (MWU- tests P < 0.05 for the latter tissues versus the former, Fig. 2). In this regard, the data show striking differences in frequency of use of the optimal codons among tissue types (Fig. 2) while the identities of optimal codons themselves are largely conserved (Additional file 1: Table S2). These patterns are consistent with a hypothesis that selection for translational optimization has been higher for genes involved in the gonads and male accessory glands, than those from the nervous system.

While few comparable data on multi-tissue expression and Fop are available, and especially with respect to sex, a study of the male-female gonads and gonadectomized tissues in *D. melanogaster* indicated that the codon usage bias was lower in male than female genes [37]. This pattern may be due to Hill-Robertson interference arising from adaptive evolution at linked amino acid sites in the males, dragging slightly deleterious codon mutations to fixation [37]. However, we found an opposite pattern in the mosquito *Aedes aegypti* where optimal codon use was higher in male than in female gonads [11]. Our results here, using four discrete paired male-female tissue types, suggest that the only sex-related difference in Fop for *G. bimaculatus* is for the somatic reproductive system (where male genes had lower Fop than female genes, Fig. 2). Thus, outside the somatic reproductive system, our data show that tissue type of maximal expression plays the predominant role in shaping Fop in this cricket model, rather than sex. Moreover, the relatively low Fop observed in the brain (Fig. 2) suggests that Hill-Robertson effects may be greatest in this tissue type, a notion that is consistent with recent observations of a rapid rate of protein sequence evolution of sex-biased brain genes in this species [66]. It is worth noting that the finding that the degree of optimal codon use is particularly pronounced for genes transcribed in the gonads in Fig. 2 may suggest greater absolute (but not relative) tRNA abundances of the optimal codons in those reproductive tissues, which are essential for formation of the sex cells.

### Functional roles of optimal and non-optimal codons inferred by their relationships to tRNA gene copies

The hypothesis of translational selection for efficient and/or accurate translation in an organism has been thought to be substantiated by associations between optimal codon use in highly expressed genes and their matching tRNA gene copy numbers in the genome [3, 5, 12, 20, 21, 23, 27–31]. In some organisms, however, the correspondence between optimal codon use in highly expressed genes and the matching tRNA abundance has been weak [23], or not observed for some codons [91, 92], which has been interpreted as limited/absent support for adaptation of tRNA abundance and optimal codon use in certain systems [23, 92]. However, growing evidence suggests that there is a complex supply-demand relationship between codons and tRNAs that may affect multiple aspects of translation [45–47, 93], such that a universal connection between optimal codons and matching tRNA gene copy numbers may not always be expected even under a selection model [20, 45, 47]. For instance, some optimal codons may obligately require wobble tRNAs (no direct matching tRNAs) [20], which act to allow slow translation [51, 52], and thus a positive relationship between codon use in highly expressed genes and high tRNA abundance would not be expected for those codons. In turn, while non-optimal (or rare) codons may have few tRNAs, and thus act to slow translation [47], in some cases they may have numerous matching tRNAs, which could conceivably allow for translational upregulation of gene mRNAs using those codons [20, 48]. Given this context, to allow a precise interpretation of the codon-tRNA relationships in Table 1, and given some variation in terminology in the literature, we explicitly describe the codons using their ΔRSCU status and their tRNA abundances as follows: Opt-codon_↑tRNAs_ are those optimal codons (elevated use in highly expressed genes) that have relatively high tRNA gene copy numbers; Opt-codon_wobble,_ include those optimal codons obligately requiring the use of wobble tRNAs; Nonopt-codon_↓tRNAs_ are the non-optimal codons (least used in highly expressed genes) with few tRNAs; and Nonopt-codon_↑tRNAs,_ represents non-optimal codons with abundant tRNA gene copies [20].

To assess the relationships between the codon use and tRNA gene numbers for each amino acid in Table 1, we first determined the number of tRNA genes per amino acid in the *G. bimaculatus* genome using tRNA-scan-SE [69, 94]. We report 1,391 putative tRNAs for the *G. bimaculatus* genome (Table 1). To evaluate the propensity for translational selection per se, defined as a strong relationship between optimal codon use in highly expressed genes and tRNAs [5, 12, 20, 23, 25], we compared the 18 primary optimal codons to the number of tRNAs per gene. We found that for 11 of 18 amino acids, the primary optimal codon had the highest or near highest matching number of tRNAs gene copies (≥18 tRNA copies) among the synonymous codons (Table 1), or Opt-codon_↑tRNAs_ status. Thus, this concurs with a model of translational selection for accurate and/or efficient translation for a majority of optimal codons in this cricket (Table 1) [5, 12, 20, 23, 25]. However, some optimal codons obligately required a wobble tRNA, or had Opt-codon_wobble_ status, which we suggest may also serve important functional roles.

#### Some optimal codons require wobble tRNAs

Seven of the 18 identified optimal codons in Table 1 had Opt-codon_wobble_ status, and had no exact matching tRNAs in the genome. These included the codons AAT (Asn), GAT (Asp), TGT (Cys), GGT (Gly), CAT (His), TTT (Phe), and TAT (Tyr) (Table 1). Thus, the elevated use of codons with Opt-codon_wobble_ status in highly transcribed genes cannot be ascribed to translational selection per se. We suggested in a recent report for *T. castaneum* that optimal codons obligately using wobble tRNAs may likely be employed in highly expressed genes as a mechanism to slow translation, perhaps for protein folding purposes [20]. Indeed, experimental research in various eukaryotic models has shown that ribosomal translocation along the mRNA is slowed by codons requiring wobble tRNAs [45, 51, 52], and thus may allow co-translational protein folding. The inefficiency of wobble interactions between codons and tRNAs, including chemically modified wobble tRNAs (e.g., adenosine to inosine, I34) in the anticodon loop [70, 71] appears to act as a mechanism to decelerate translation as compared to codons with exact tRNA matches [45, 46]. In this regard, wobble codons in highly expressed genes studied here may serve a similar function to non-optimal codons (those that have few tRNAs, see below section), which growing studies suggest may regulate the rate, or rhythm, of translation to allow co-translational protein folding [47, 53–56]. Notably, we found the highly transcribed genes studied in *G. bimaculatus* were preferentially involved in protein folding as shown in Table 2, and thus this comprises a primary active process within the tissues/cells under study. In this regard, our collective results suggest a hypothesis that wobble codons in highly transcribed genes may slow translation and effectively assist in the process of protein folding.

To further study the possible roles of wobble codons, we assessed the gene ontology (GO) functions of the four codons with Opt-codon_wobble_ status that had the highest ΔRSCU values (GGT, GAT, CAT and TAT with ΔRSCU values of + 0.610, + 0.520, + 0.511 and + 0.430 respectively (Table 1)) to determine if genes using these codons tended to be involved in particular processes. For this, we examined the subset of highly expressed genes that were enriched for each wobble codon (favored use indicated by RSCU≥1.5, whereas a value of 1 would indicate equal use of the codon per codon family) in the organism-wide dataset (Table 1), and for the genes with Top5_One-tissue_ status in the gonads (Additional file 1: Table S2), which had the largest N values of genes of any tissue type (Additional file 1: Table S2; ontology was ascertained from putative orthologs to *D. melanogaster* (e < 10^− 3^, BLASTX [73]), see Methods). The results are shown in Additional file 1: Table S3. The functions of the organism-wide highly expressed genes with especially elevated use of the Opt-codon_wobble_ codons included ribosomal protein genes, and genes involved in mitochondrion functions (Additional file 1: Table S3), thereby specifically affirming that high use of the wobble codons are apt to serve functions in these types of genes (Table 2). For the gonads, we found that the top GO clusters for genes with elevated use of GAT that were expressed in the ovaries (with Top5_One-tissue_ status) and of TAT in the testes (with Top5_One-tissue_ status) were involved in mitosis and cell cycle functions (Additional file 1: Table S3). Thus, this pattern for highly expressed gonadal genes in this cricket is in agreement with a prior experimental study that suggested the use of wobble codons in genes in cultured human and yeast cells might regulate the cell cycle by controlling translation of cell-cycle genes [95]. Taken together, our results are suggestive that the use of Opt-codon_wobble_ codons in highly expressed cricket genes may act to slow translation as a means to regulate the level of cellular proteins, and to ensure proper co-translational folding, particularly affecting genes involved in the cell cycle (Additional file 1: Table S3) and ribosomal and mitochondrial proteins (Table 2).

#### Non-optimal codons may have different functions that depend on tRNA abundance

The primary non-optimal codon per amino acid was defined as the codon with the largest negative ∆RSCU with a statistically significant *P* value [20]. With respect to the identified non-optimal codons, we found striking patterns with respect to tRNAs that concur with two possible functional roles, that include firstly, slowing translation, and secondly, regulating differential translation of cellular mRNAs. With respect to the former case, we found two amino acids had a primary non-optimal codon with Nonopt-codon_↓tRNAs_ status, that included CGC (Arg), ATC (Ile) (Table 1). This suggests their infrequent use in highly expressed genes may be due to the rarity or absence of matching tRNAs in the cellular tRNA pools. Moreover, these codons were not only non-optimal, and thus by definition are rare in highly transcribed genes, but their exact matching tRNAs were absent in the genome, and thus require wobble tRNAs, a combination that would in theory make them especially prone to slowing down translation. The use of non-optimal codons has been suggested to decelerate translation, which may prevent ribosomal jamming [26], and/or permit proper protein folding [47, 53, 54, 96], while, as described above, the use of codons requiring wobble tRNAs may also slow translation [45, 51, 52]. Thus, we propose the use of these two codons in genes that have Nonopt-codon_↓tRNAs_ status, and require wobble tRNAs, could play significant roles in slowing translation in highly expressed genes in *G. bimaculatus*.

Importantly however, the other non-optimal codons in Table 1 had tRNA counts markedly higher than zero (≥15 gene copies; Nonopt-codon_↑tRNAs_ status). Thus, the infrequent use of those non-optimal codons in the highly expressed genes is not likely to be due to a role in slowing translation. In fact, the use of these codons combined with high tRNA abundance suggests the potential for a high supply:demand ratio [20, 45, 48–50], a relationship that may give rise to preferential translation of any highly expressed genes that contain unusually elevated Nonopt-codon_↑tRNAs_ codons [20]. This proposed mechanism of up-translation using non-optimal (or rare) codons has been recently suggested for stress genes in yeast [48], and for highly expressed genes in the red flour beetle, wherein genes with an elevated frequency of Nonopt-codon_↑tRNAs_ status codons were linked to specific biological functions [20], suggesting their mRNAs may be preferentially translated. In this regard, the Nonopt-codon_↑tRNAs_ status codons in *G. bimaculatus* could also have significant biological roles in up-regulation of specific cellular mRNAs in this cricket model.

To further evaluable this possibility for *G. bimaculatus*, we studied as examples the Nonopt-codon_↑tRNAs_ codon GTG for Val, which had an organism-wide ΔRSCU of − 0.484 and 40 tRNAs, the codon GGC for Gly with respective values of − 0.709 and 41 tRNAs (note both Val and Gly are four-fold degenerate), and CTG for the six-fold degenerate Leu with a ΔRSCU of − 0.692 and 30 matching putative tRNAs (Table 1). These were chosen as examples due to their relatively high putative tRNA counts (as compared to other Nonopt-codon_↑tRNAs_ codons from amino acids with the same degeneracy level). For each of these codons, we examined those Top5_One-tissue_ genes (only in the top 5% expression in one tissue type) in the gonads that had RSCU value ≥1.5, indicating enhanced use. The results are shown in Table 3. We found that genes preferentially using Nonopt-codon_↑tRNA_ codons were associated with a diverse range of functions. For example, for the ovaries, the highly expressed genes that preferentially used the Nonopt-codon_↑tRNAs_ codon GTG (for Val) included a match to *Bicaudal C* (*BicC*), which is involved in oogenesis [98]. Remarkably, this ovary gene also had elevated use of the codons GGC ad CTG (Table 3). Further, for the ovaries, a gene matching *santa-maria*, which has been associated with phototransduction [99] and apoptosis [100], had elevated use of each of the wobble codons GTG, GGC and CTG. The fact that genes matching *BicC* and *santa-maria* each had high use of all three of these Nonopt-codon_↑tRNAs_ codons, which by definition have abundant matching tRNA genes, suggests their gene transcripts may be preferentially translated in the ovary as compared to other transcripts in the transcript pool. For CTG (Leu), the Top5_One-tissue_ genes in the ovaries preferentially using this codon with Nonopt-codon_↑tRNAs_ status included another apoptosis gene, *apoptosis inducing factor* (*AIF*) [101], which also had elevated use of GGC for Gly, suggesting these codons may facilitate apoptosis in the female gonad cells. With respect to the testis, GTG (Val) was preferentially used in genes such as *belle*, which is involved in male germ-line stem cell development [102, 103] and *no child left behind* (*nclb*), involved in male gonad development [104], suggesting that use of this non-optimal codon may promote translation of these particular transcripts in the male gonadal mRNA pools. Enhanced use of GGC and CTG in testes was found for genes matching *Dual-specificity tyrosine phosphorylation-regulated kinase 2* (*Dyrk2*), which is involved in apoptosis and sensory roles [105, 106], and *short spindle 3 (ssp3)*, involved in male meiosis [107] (Table 3), infers that these two codons may promote translation of apoptosis and meiotic proteins in the testes. When taken together, these patterns in *G. bimaculatus*, similar to recent findings in *T. castaneum* [20], suggest that the combination of elevated use of non-optimal codons and a high supply of tRNAs may plausibly be involved in preferential translation of the transcripts of specific genes in this system, particularly for apoptosis genes and genes with female and male gonadal functions (Table 3).

**Table 3:** Examples of genes that exhibit the top 5% expression levels in the ovaries and top 5% expression levels in the testes (but are not in the top 5% of any other tissue type, Top5_One-tissue_) in *G. bimaculatus* that have elevated use of a non-optimal codon with high tRNAs counts (Nonopt-codon_↑tRNAs_ status; elevated use in this table indicates the RSCU in a gene is ≥1.5). The codons include GTG for Val, GGC for Gly, and CTG for Leu (RSCU values ≥1.5). Genes are listed that have an identified putative *D. melanogaster* (Dmel) ortholog (best match BLASTX e < 10^−3^ [73] and a known gene name at FlyBase [97]

GB ID	Dmel ID	Gene Name
**Ovaries- GTG for Val (RSCU ≥ 1.5)**		
GBI_17906-RA	FBgn0039889	*ADP ribosylation factor-like 4* (*Arl4*)
GBI_01735-RA	FBgn0261788	*Ankyrin 2* (*Ank2*)
GBI_16610-RA	FBgn0024227	*aurora B* (*aurB*)
GBI_20301-RA	FBgn0000182	*Bicaudal C* (*BicC*)
GBI_10942-RA	FBgn0024491	*Bicoid interacting protein 1* (*Bin1*)
GBI_05907-RA	FBgn0000337	*cinnabar* (*cn*)
GBI_11302-RA	FBgn0030608	*Lipid storage droplet-2* (*Lsd-2*)
GBI_09650-RA	FBgn0031145	*Nuclear transport factor-2* (*Ntf-2*)
GBI_06633-RB	FBgn0031530	*Polypeptide GalNAc transferase 2* (*Pgant2*)
GBI_13292-RA	FBgn0039214	*puffyeye* (*puf*)
GBI_11680-RC	FBgn0004855	*RNA polymerase II 15kD subunit (RpII15*)
GBI_13051-RB	FBgn0025697	*scavenger receptor acting in neural tissue and majority of rhodopsin is absent* (s*anta-maria*)
GBI_03901-RD	FBgn0003312	*shadow* (*sad*)
GBI_03557-RA	FBgn0037802	*Sirtuin 6* (*Sirt6*)
GBI_00841-RB	FBgn0003714	*technical knockout* (*tko*)
**Testes- GTG for Val (RSCU ≥ 1.5)**		
GBI_00920-RA	FBgn0038984	*Adiponectin receptor* (*AdipoR*)
GBI_00615-RA	FBgn0263231	*belle* (*bel*)
GBI_03558-RA	FBgn0032820	*fructose-1,6-bisphosphatase* (*fbp*)
GBI_04579-RA	FBgn0030268	*Kinesin-like protein at 10A* (*Klp10A*)
GBI_09377-RA	FBgn0015754	*Lissencephaly-1* (*Lis-1*)
GBI_12141-RA	FBgn0038167	*Lkb1 kinase* (*Lkb1*)
GBI_02406-RA	FBgn0263510	*No child left behind* (*nclb*)
GBI_09426-RA	FBgn0261588	*pou domain motif 3* (*pdm3*)
GBI_08602-RA	FBgn0036257	*Rho GTPase activating protein at 68F* (*RhoGAP68F*)
GBI_05329-RA	FBgn0032723	*short spindle 3* (*ssp3*)
**Ovaries- GGC for Gly (RSCU ≥ 1.5)**		
GBI_17906-RA	FBgn0039889	*ADP ribosylation factor-like 4(Arl4)*
GBI_06216-RA	FBgn0031392	*Apoptosis inducing factor (AIF)*
GBI_20301-RA	FBgn0000182	*Bicaudal C (BicC)*
GBI_11302-RA	FBgn0030608	*Lipid storage droplet-2 (Lsd-2)*
GBI_05398-RA	FBgn0029687	*VAMP-associated protein of 33 kDa ortholog A(Vap-33A)*
GBI_09822-RD	FBgn0261458	*capulet (capt)*
GBI_01828-RA	FBgn0011296	*lethal (2) essential for life (l(2)efl)*
GBI_10179-RA	FBgn0024841	*pterin-4a-carbinolamine dehydratase (pcd)*
GBI_13051-RB	FBgn0025697	*santa-maria*
**Testes- GGC for Gly (RSCU ≥ 1.5)**		
GBI_15155-RA	FBgn0016930	*Dual-specificity tyrosine phosphorylation-regulated kinase 2* (*Dyrk2*)
GBI_09377-RA	FBgn0015754	*Lissencephaly-1(Lis-1)*
GBI_00388-RA	FBgn0010288	*Ubiquitin carboxy-terminal hydrolase (Uch)*
GBI_09426-RA	FBgn0261588	*Pou domain motif 3 (pdm3)*
GBI_05329-RA	FBgn0032723	*short spindle 3 (ssp3)*
**Ovaries- CTG for Leu (RSCU ≥ 1.5)**		
GBI_17906-RA	FBgn0039889	*ADP ribosylation factor-like 4* (*Arl4*)
GBI_01735-RA	FBgn0261788	*Ankyrin 2* (*Ank2*)
GBI_06216-RA	FBgn0031392	*Apoptosis inducing factor* (*AIF*)
GBI_07513-RA	FBgn0005666	*bent* (*bt*)
GBI_20301-RA	FBgn0000182	*Bicaudal C* (*BicC*)
GBI_05907-RA	FBgn0000337	*cinnabar* (*cn*)
GBI_11302-RA	FBgn0030608	*Lipid storage droplet-2* (*Lsd-2*)
GBI_16524-RA	FBgn0027786	*Mitochondrial carrier homolog 1* (*Mtch*)
GBI_09650-RA	FBgn0031145	*Nuclear transport factor-2* (*Ntf-2*)
GBI_05851-RA	FBgn0003074	*Phosphoglucose isomerase* (*Pgi*)
GBI_06633-RB	FBgn0031530	*Polypeptide GalNAc transferase 2* (*pgant2*)
GBI_09582-RA	FBgn0036187	*RIO kinase 1* (*RIOK1*)
GBI_13051-RB	FBgn0025697	*santa-maria*
GBI_03901-RD	FBgn0003312	*shadow* (*sad*)
**Testes- CTG for Leu (RSCU ≥ 1.5)**		
GBI_00369-RA	FBgn0003884	*Alpha-Tubulin at 84B* (*alphaTub84B*)
GBI_15155-RA	FBgn0016930	*Dyrk2*
GBI_03558-RA	FBgn0032820	*fructose-1,6-bisphosphatase* (*fbp*)
GBI_10438-RA	FBgn0038923	*mitochondrial ribosomal protein L35* (*mRpL35*)
GBI_09426-RA	FBgn0261588	*Pou domain motif 3* (*pdm3*)
GBI_08602-RA	FBgn0036257	*Rho GTPase activating protein at 68F* (*RhoGAP68F*)
GBI_05329-RA	FBgn0032723	*short spindle 3* (*ssp3*)
GBI_00450-RA	FBgn0024289	*Sorbitol dehydrogenase 1* (*Sodh-1*)
GBI_14282-RA	FBgn0029763	*Ubiquitin specific protease 16/45* (*Usp16–45*)

### Amino acid use, biosynthesis costs, and tRNA gene copies have interdependently evolved

**Fig. 3:**
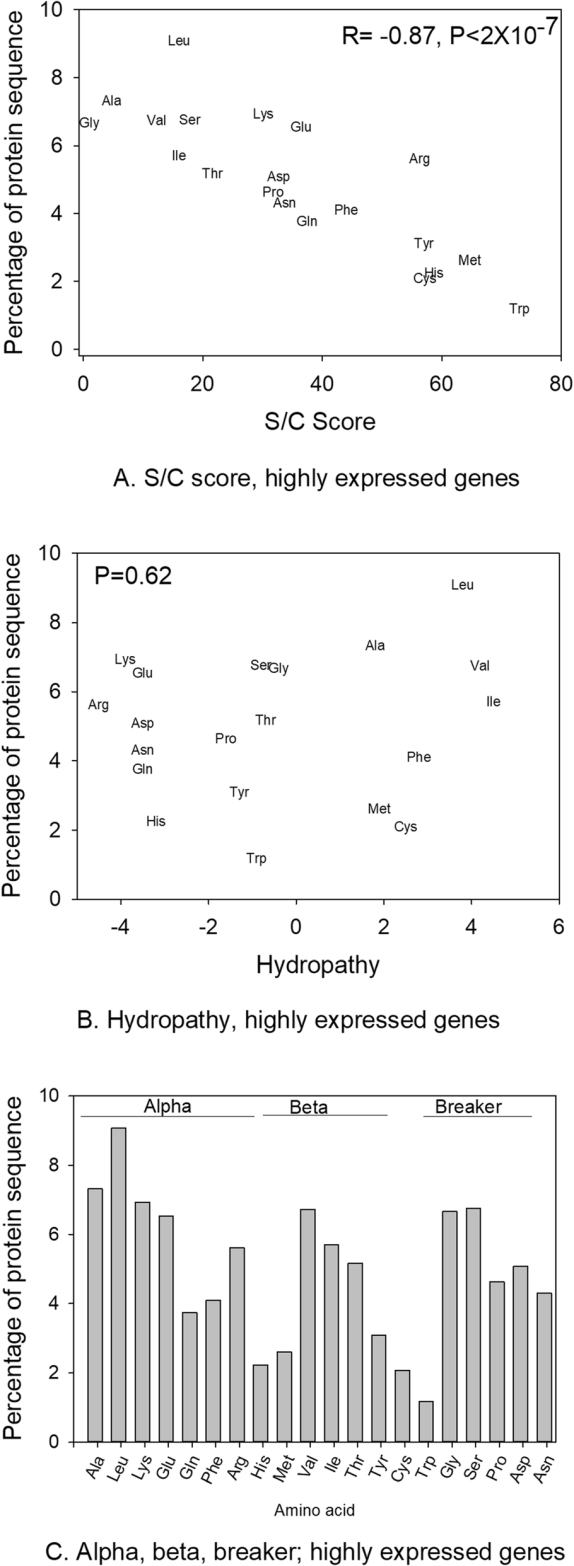
The relationship between amino acid properties and amino acid use (percent per gene, averaged across genes) in the organism-wide highly expressed genes. **a** size/complexity (S/C) score; **b** hydropathy, and **c** folding properties. For **a** and **b** Spearman’s R and/or *P* values are shown, and for **c** no differences were detected between groups (alpha, beta, and breaker, Ranked ANOVA P > 0.05)

Next, we asked whether amino acid use in the highly expressed genes in *G. bimaculatus* (top 5% using the organism-wide assessment) varied with their size/complexity (S/C) scores, which were developed to quantify the relative biosynthesis costs of different amino acids [58], hydropathy, or with their broad role in protein folding properties [108, 109] (Additional file 1: Table S4). As shown in Fig. 3, for highly expressed genes the amino acid usage (across all 20 amino acids) was not correlated to hydropathy (Spearman’s correlation across all 777 organism-wide highly expressed genes *P* > 0.60) and showed no broad relationship to specific protein folding properties (ranked ANOVA *P* > 0.05 between groups, Fig. 3bc). However, a very strong negative correlation was observed between amino acid use and S/C scores across the 20 amino acids (Spearman’s R = -0.87, *P* < 2X10^− 7^, Fig. 3a, Table 4; see also [10]). An inverse relationship between S/C score and the frequency of the 20 amino acids was also observed across all 15,539 studied *G. bimaculatus* genes irrespective of expression level (for all genes R = -0.70, *P* = 4X10^− 4^, Additional file 1: Fig. S1), but the correlation was stronger in the subset of highly expressed genes, suggesting that the connection between amino acid use and S/C scores is ameliorated with elevated transcription. Thus, these patterns both at the genome-wide level and using highly expressed genes measured across nine tissue types, indicate preferential use of low-cost amino acids in genes producing abundant mRNAs.

**Table 4:** The average amino acid use of the top 5% expressed genes (Top5_One-tissue_) in *G. bimaculatus* and 5% lowest expressed genes for the organism-wide analyses (using average expression across all nine tissue types). The number of predicted tRNAs in the genome per amino acid are shown. SE is the standard error

Amino acid (AA)	S/C Score	AA Freq. High exp	SE	AA Freq. Low exp	SE	Percent Diff.	P	tRNAs
Gly	1	6.66	0.21	8.71	0.13	**−30.70**	**	71
Ala	4.76	7.32	0.24	11.54	0.14	**−57.72**	**	75
Val	12.28	6.73	0.19	6.27	0.08	**+ 6.80**	**	96
Ile	16.04	5.70	0.15	2.91	0.04	**+ 49.01**	**	41
Leu	16.04	9.07	0.26	8.13	0.10	**+ 10.31**	**	141
Ser	17.86	6.75	0.21	7.63	0.11	**−12.94**	**	132
Thr	21.62	5.16	0.15	5.08	0.07	+ 1.69		103
Lys	30.14	6.93	0.18	3.53	0.06	**+ 49.08**	**	70
Pro	31.8	4.62	0.15	6.95	0.11	**−50.40**	**	103
Asp	32.72	5.08	0.16	3.83	0.06	**+ 24.64**	**	31
Asn	33.72	4.30	0.13	2.68	0.04	**+ 37.70**	**	37
Glu	36.48	6.53	0.22	5.09	0.07	**+ 22.08**	**	49
Gln	37.48	3.75	0.15	3.49	0.05	**+ 6.92**	*	76
Phe	44	4.10	0.10	2.70	0.04	**+ 34.20**	**	48
Arg	56.34	5.61	0.15	10.04	0.12	**−78.95**	**	125
Tyr	57	3.10	0.08	1.87	0.05	**+ 39.53**	**	43
Cys	57.16	2.08	0.06	2.51	0.03	**−20.75**	**	38
His	58.7	2.24	0.07	2.53	0.04	**−12.99**	**	37
Met	64.68	2.61	0.06	2.32	0.02	**+ 10.93**	**	43
Trp	73	1.18	0.03	1.48	0.02	**−25.80**	**	32

Notes: A negative correlation between S/C score and the frequency of amino acids was observed for highly and lowly expressed genes (Spearman’s Ranked R = -0.87 and − 0.75, P < 10^−7^). Further, a positive correlation between the frequency of amino acids and tRNA counts was observed for highly and lowly expressed genes (Spearman’s Ranked R = 0.65 and 0.74, P = 2.6X10^−3^ and P < 10^− 7^). Percent Diff. = percent difference. In column with P values, * indicates *P* < 0.05–0.001, **indicates *P* < 0.001 using a two tailed t-test (α = 0.05). All P values for t-tests in the table withstand Bonferroni correction (P < 0.05 after corrected by the number of tests) with the exception of Gln

To further decipher this relationship, we compared amino acid usage using the organism-wide highest and lowest expressed genes (top and lowest 5%, averaged across nine tissues). As shown in Table 4, we found that 19 of 20 amino acids had a statistically different frequency between the most and least transcribed genes in the genome (all t-tests *P* < 0.05), with the only exception being Thr (and Gln when using the Bonferroni correction). The amino acids with the largest increase in frequency in highly expressed genes (as compared to lowly expressed) were Ile (S/C score = 16.04; with 49.0% greater use under high expression) and Lys (30.14; 49.1% greater use under high expression), suggesting that enhanced use of these amino acids with intermediate S/C scores may be more crucial to efficient translation or function of abundant transcripts than the use of those with the lowest possible S/C scores in this taxon. We note this is consistent with an earlier analysis based on a partial transcriptome from one pooled ovary/embryo sample and without tRNA data in that study, where amino acids with intermediate S/C scores Glu, Asp, and Asn were preferred [10], which all had > 22% increased use under high transcription here. This type of complex relationship between S/C score and amino acid use has also been suggested in spiders [57].

Under a null hypothesis of equal usage of each of 20 amino acids, we would assume a frequency of 5% for every amino acid per gene, with values above and below this threshold indicating favored and unfavored usage respectively. In this context, we observed that for the five highest cost amino acids (Tyr, Cys, His, Met and Trp, S/C scores of 57.00 to 73.00), the average usage was less than 5% (between 1.18 and 3.10%) in both the highly and lowly expressed genes (Table 4), indicating these biochemically costly amino acids are consistently rarely used in this taxon. Taken together, organism-wide highly expressed genes in *G. bimaculatus* exhibit a pattern of elevated use of amino acids with low S/C scores (Fig. 3a), and also exhibit a tendency for elevated use of specific amino acids with intermediate S/C scores (Table 4), and very low use of the highest cost amino acids. We speculate that the pattern of favored use of some intermediate cost amino acids may be due to the roles of these amino acids in protein folding (e.g., beta and alpha folding respectively, Additional file 1: Table S4) and thus their use may ensure proper function of abundantly produced gene products.

With respect to tRNA abundances, we found that amino acid frequencies in Table 4 were positively correlated to the tRNA gene counts per amino acid (the tRNA counts included all those matching any of synonymous codons per amino acid) in *G. bimaculatus*. The correlation was observed both for the highly and for the lowly expressed genes (Spearman’s Ranked R = 0.65 and 0.75, *P* = 2.6X10^− 3^ and *P* < 10^− 7^, Table 4). Thus, this suggests the frequency of amino acid use within genes is connected to its tRNA abundance in this organism. However, despite being correlated in both groups (high and low expressed genes) in this cricket species, we suggest that the relationship is apt to be most beneficial to the organism by reducing the translational costs of genes that are highly transcribed, as these genes should presumably be most commonly translated.

**Fig. 4:**
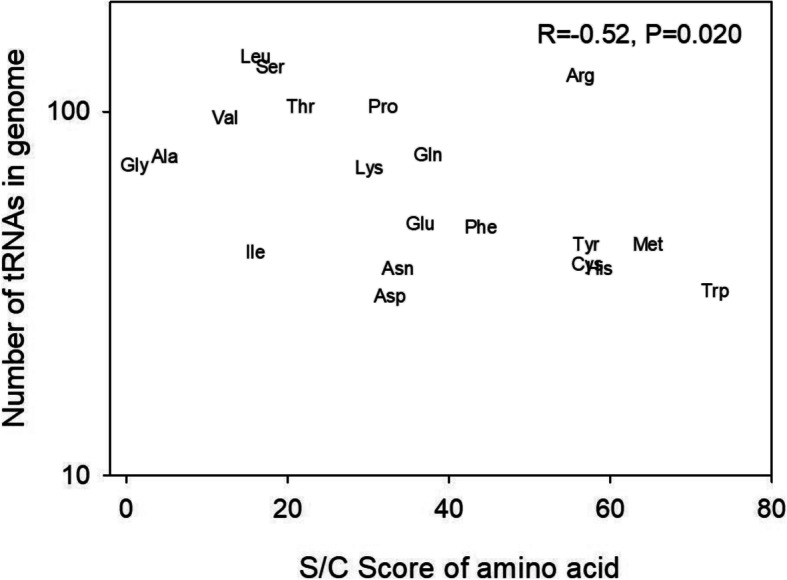
The predicted gene counts of tRNAs in the *G. bimaculatus* genome and the S/C score of each of 20 amino acids [58]. The Spearman R correlation and P values are shown

We next asked whether tRNA abundance, or gene copy number, was connected to S/C scores in *G. bimaculatus*. Indeed, the S/C scores of the 20 amino acids showed a tendency to be inversely connected to the total tRNA counts per amino acid in the organism-wide highly expressed genes (Spearman’s R = -0.52, *P* = 0.02, Fig. 4). Thus, the abundance of tRNAs in the genome is directly connected to how biochemically costly an amino acid is to produce by the organism. While comparable studies of relationships between biosynthetic amino acid costs and tRNAs are uncommon, a similar negative pattern has been observed in a study from beetles [23], suggesting this phenomenon may be shared among diverse insects. Taking all our results in combination, it is evident that amino acid frequency is positively correlated to the matching tRNA gene counts (Table 4) and negatively correlated to S/C scores (Fig. 3a, Additional file 1: Fig. S1), and that tRNA gene counts per amino acid are negatively related to S/C scores (Fig. 4). In other words, genes exhibit a tendency for preferred use of low-cost amino acids that have abundant tRNAs. We therefore suggest the hypothesis that all three parameters, amino acid frequency, tRNA genes in the genome, and biochemical costs, have evolved interdependently for translational optimization in *G. bimaculatus.*

It should be noted that while we specify herein that our tRNA counts obtained from tRNA-scan-SE (v. 2.0.5) [69, 94] from the recently available cricket genome [67] are considered preliminary predictions in this study (see Methods, Table 1), the accuracy of this list is substantiated by the marked correlation of tRNA gene counts with S/C scores (Fig. 4) and with amino acid frequency (Table 4). In this regard, we consider the relative tRNA counts apt to provide an appropriate and accurate profile for *G. bimaculatus*.

#### Variation in amino acid use with respect to sex and tissue type

Finally, we determined whether amino acid frequency per gene varied among tissue type or sex for those genes with Top5_One-tissue_ status. The results for amino acid frequency are shown in Additional file 1: Table S5, and correlations between use for each sex per tissue type are provided in Additional file 1: Table S6. For each sex, we found strong correlations in the frequency of amino acid use (across 20 amino acids) for all paired contrasts of tissues, with Spearman R values between 0.861 and 0.98 (*P* < 2X10^− 6^). This suggests the relative amino acid use is largely consistent among highly expressed genes from all tissue types. However, the R values were weakest (R < 0.9) for contrasts of the male gonad to all other tissues, suggesting a possible testis-effect on amino acid use. In terms of differences between sexes, we determined the percent difference in frequency of amino acid use between females and males for each tissue type (Additional file 1: Table S5). We found that amino acid use varied between the sexes, with between two to six amino acids per tissue type (gonad, somatic reproductive system, brain, ventral nerve cord) exhibiting statistically significant differences between sexes. As an example, for the Top5_One-tissue_ genes from the brain which had six amino acids with statistically significant differences between males and females, we found that some amino acids, namely Arg and Tyr, had in excess of 21% difference in their use between the sexes in *G. bimaculatus* (t-test *P* = 0.007 and 0.017 respectively; Additional file 1: Table S5), thus suggesting particularly marked variation for this tissue. In this regard, there are non-negligible differences in amino acid use between the sexes, particularly for the brain, suggesting that high expression in a particular sex may be a significant factor contributing to amino acid use.

## Discussion

Taken together, the present results provide several lines of evidence suggesting adaptation of codons and amino acids to their matching tRNAs in *G. bimaculatus*. These include firstly showing that optimal codons are well correlated to tRNA gene copy numbers (Table 1), secondly showing that when we consider all tRNAs that encode a single amino acid (summing tRNAs across all synonymous codons per amino acid) there was a positive correlation to amino acid use in genes (Table 4), and thirdly revealing that tRNA gene copy numbers per amino acid were inversely correlated to the size/complexity scores of amino acids (Fig. 4). These various and well supported correlations are consistent with a model whereby selection had favored a codon use-tRNA relationship and an amino acid use-tRNA relationship in this cricket (and thus suggest rejection of the null hypotheses of no relationships).

Further, with respect to codon use, using small intervals of intron AT content (0.1) to control for background pressures such as mutation or BGC [7, 79], we found evidence of elevated AT3 codons under high versus low transcription (Fig. 1), and the consistent use of specific favored codons under high expression (Table 1), which in itself concurs with a model of translational selection [2, 7, 9, 10, 19, 20, 22, 68]. Thus, while non-adaptive forces such as mutational biases and BGC may influence genome-wide codon use in this species, our cumulative evidence indicates that in its most highly expressed genes, adaptative processes have at least partly contributed to optimal codon use.

A recent study by Gaultier et al. 2018 [85] suggested that translational selection favoring optimal codon use in highly expressed genes may generally be weak or absent in large vertebrates, including mammals, whereby codon use may be largely influenced by mutation and/or BGC [85, 92, 110, 111] (but not always [79, 85]). In turn, translational selection for optimal codons in highly expressed genes may be more apt to be found in organisms with larger populations (4N_e_s > 1, where N_e_ = effective population size, s = selection coefficient), including solitary (non-social) insects [85], such as *G. bimaculatus* studied here [112]. In this regard, it may be unsurprising that evidence available in certain mammals suggests a poor signal of expression-related adaptation between codon use and matching tRNA pools in those systems [92, 110], as there is likely weak or absent translational selection. However, translational selection and thus codon-tRNA relationships may be much more likely to occur in crickets, as we suggest here, similar to other solitary insects such as *D. melanogaster* (flies) [19, 22] and *T. castaneum* (beetles) [20]. Our results extend beyond those relationships, and further suggest that codons with other types of statuses in highly expressed genes, namely Opt-codon_wobble,_ Nonopt-codon_↓tRNAs,_ and Nonopt-codon_↑tRNAs_ have potentially evolved for specific roles in controlling translational rates and/or protein levels in this cricket.

## Conclusions

Herein, we have studied codon and amino acid use in a cricket model system and proposed a significant role of selection within its most highly transcribed genes, at the organism-wide level (Table 1) and in different tissue types (Additional file 1: Table S2). Future research should include the direct quantification of tRNAs in different tissue types [39, 48, 74, 113], to assess whether those results add support to the conclusion of similar relative tRNA abundances across tissue type and sex in this cricket. Such an approach will also help discern why this cricket species may have less propensity for tissue-related optimal codons than other organisms studied to date [20, 36, 38, 40]. While our data suggest that mutational AT biases may partly contribute towards genome-wide codon use patterns in *G. bimaculatus*, and we do not exclude a role of BGC in the variation in GC/AT content among genes, the collective patterns are consistent with the hypothesis that translational selection significantly contributes to optimal codon use under high transcription. Further studies should rigorously evaluate the possible roles of BGC in codon use in this cricket species [85], including approaches that consider meiotic recombination rates, expression level in meiotic cells, and their relationships to GC (and thus AT) content (cf. [83, 114, 115]), as more genomic, population data, and recombination data begin to emerge in this taxon.

Another meaningful direction for future study may include the identification of ramping of codons in CDS [116], which may cause a slow-down in translation, particularly at the beginning of CDS, and may potentially increase translational efficiency downstream of the ramp [26, 45, 51, 52, 116]. In particular, ramps using the codons with Nonopt-codon_↓tRNAs_ and Opt-codon_wobble_ status identified herein (Table 1) are candidates to play roles in regulating translation elongation rates using ramping in CDS, and may vary with high versus low expression. In addition, recent research suggests codon use and hydrogen bonding ramps may have roles in dsDNA unwinding and transcriptional regulation, as inferred in Bacteria and Archaea (but not Fungi) [117], and thus this also provides a meaningful avenue for further study in this cricket model and other multicellular animals. Finally, further studies should be conducted of the frequencies of optimal, as well as non-optimal, codons and their relationships to tRNA abundances and gene functionalities in a wider range of multicellular organisms. Such research will reveal whether the phenomena observed herein are shared across divergent systems.

## Methods

### Biological samples and RNA-seq

*Gryllus bimaculatus* cultures were established from animals originally obtained from Livefoods Direct (Sheffield, UK) and maintained as an inbred laboratory colony for 15 years, as previously described [118]. RNA-seq was obtained for four adult male and female tissue types, the gonad (testis for males, ovaries for females), somatic reproductive system, brain and ventral nerve cord for each of two females and two males, and for the male accessory glands (Additional file 1: Table S1) [66]. Gene expression level was determined for all 15,539 *G. bimaculatus* annotated protein-coding genes (CDS, longest CDS per gene) [67] that had a start codon and were > 150 bp. The expression level of each *G. bimaculatus* gene was determined by mapping trimmed reads per RNA-seq dataset per tissue to the complete CDS list using Geneious Read Mapper [119] to determine FPKM per gene. FPKM was robust to mapping programs, and other common mappers including BBmap (https://jgi.doe.gov/data-and-tools/bbtools/bb-tools-user-guide/bbmap-guide/) and Bowtie2 [120] yielded similar results [66].

For each CDS, the relative synonymous codon usage (RSCU) was assessed for each amino acid with synonymous codons, whereby RSCU values > 1 and < 1 respectively indicate greater and lower use of a synonymous codon than that expected under equal codon use, and elevated values of codons for each amino acid indicate more frequent usage [25]. The identification of optimal and non-optimal codons was determined using ∆RSCU and was statistically assessed using t-tests of the RSCU of highly versus lowly expressed genes, which together has been supported as a stringent means to determine codon status [2, 7, 9, 10, 15, 19, 20]. For the organism wide analyses (Table 1), for each codon this was calculated as follows: ∆RSCU = RSCU_Mean Highly Expressed CDS_-RSCU_Mean Lowly Expressed CDS_, where RSCU_Mean Highly Expressed CDS_ = the mean RSCU for the genes with the highest 5% of average expression (across nine tissue types) among all 15,539 genes and RSCU_Mean Lowly Expressed CDS_ = mean RSCU for genes with the lowest 5% expression, including all those with tied FPKM values at the cutoff.

To isolate the effect of high expression in each individual tissue type, the optimal codon statuses were determined separately for each of the nine tissues under study (males and females for each tissue type, and male accessory glands). It has been suggested that optimal codon use in a gene largely depends on the tissue in which it is maximally expressed [20, 36]. Accordingly, to identify optimal codons for each tissue type, we examined those genes that were in the top 5% expression in that one tissue type and not in the top 5% expression for any of the remaining eight tissues (denoted as Top5_One-tissue_) versus those with the lowest 5% expression (or all those tied with the FPKM cutoff of the lowest 5% [20]). Using these subsets of highly and lowly expressed genes within each tissue, the ∆RSCU was determined for each tissue type in the same manner described for the organism-wide optimal codons.

The RSCU per codon per gene was determined in CAICAL [121] for each of the 15,539 genes under study, which was used to calculate ∆RSCU per codon using highly and lowly expressed genes. The frequency of optimal codons (Fop) [4] for each gene under study was determined, using the identified optimal codons, in the program CodonW [122]. Fop was then compared for genes with high transcription in the various tissue types in *G. bimaculatus*. For all statistical analysis, unless otherwise specified, α = 0.05, and was conducted using SYSTAT (Systat Software, San Jose, CA). Original code was not required or utilized for any analysis herein.

### Intron analysis

We compared the AT (or GC) content of introns, which are thought to largely reflect the innate mutational pressures on the nucleotide content of genes [79, 123, 124], to the AT3 content (third nucleotide position) of CDS of highly and lowly expressed genes for the *G. bimaculatus* organism-wide optimal codons [20]. For this, using the genomic data for *G. bimaculatus*, we extracted the introns for all genes (with introns), and retained those > 50 bp after trimming of 10 bp from the 5′ and 3′ ends which may contain regulatory/conserved regions [79]. For additional stringency, given that highly transcribed genes have been suggested to exhibit mutational biases (e.g., C to T) within a small number of organisms (e.g., *E. coli*, humans [81, 82]), we tested whether there was a correlation between gene expression and intron AT content in *G. bimaculatus*. To further assess the role of selection, as compared to mutation, in favoring AT3 codons (Table 1), genes from the top 5% and lowest 5% gene expression categories were placed into one of five bins based on their AT-I content as shown in Fig. 1.

### tRNA gene copies

The number of tRNA genes per amino acid in the *G. bimaculatus* genome was determined using the recently updated version of tRNA-scan-SE (v. 2.0.5) [69, 94]. The Eukaryotic filer called EukHighConfidenceFilter was used, which was designed to narrow the tRNA-scan output to a conservative high confidence tRNA [69] (used at default settings with the exception of ml − 1). We note that since the rigor of the updated program has not been explicitly tested in insects outside *Drosophila* (*P. Chan*, personal communication), we consider the tRNA predictions preliminary, and focus on the relative values of tRNAs among codons and amino acids. The accuracy of the predictions, however, is strongly supported by the correlations between tRNA gene copy numbers. amino acid costs and amino acid frequency (see Discussion). The filter acted to reduce the absolute counts of tRNAs per amino acid in the high confidence dataset. Nonetheless, the tRNA counts with and without the filter were strongly correlated across amino acids (Spearman’s Ranked R = 0.90, *P* < 2X10^− 7^), and thus relative gene counts remain intact using both measures.

### Amino acid use

Amino acid frequency per gene was determined using Geneious [119]. The frequency of each of the 20 amino acids in protein-coding genes in an organism may be influenced by factors such as their size/complexity Dufton scores (which range from 1 to 73 depending on the amino acid, [58]), as well as hydropathy (where positive hydrophobicity values indicate hydrophobic nature, while negative values suggest a hydrophilic amino acid [108, 109]), and/or their role in protein folding structures (alpha helices, beta sheets, or breakers used to affect bonding in helices) [109]. We thus aimed to study each of these parameters, using established values per amino acid shown in Additional file 1: Table S4.

### Gene ontology

For ascertaining putative gene ontology functions, we used the gene ontology from the fly *D. melanogaster*, which comprises the most well studied insect genome to date [97]. For this, we conducted a BLAST search of the full *G. bimaculatus* CDS list under study to *D. melanogaster* CDS list (version 6.29 [97]) using BLASTX [73], applying a cutoff of e < 10^− 3^. For those genes having matches within these criteria, the *D. melanogaster* gene identifiers were then input into the program DAVID [72] for gene ontology analyses and searched in FlyBase [97].

## Supplementary Information

**Additional file 1.** The file contains the Supplementary Tables, Figures and Text which are denoted and Tables S1 to S6, Figure S1, and Text File S1.

## Data Availability

All RNA-seq data under study are described in Additional file 1: Table S1 and are available at the NCBI BioProject under the project identifier PRJNA564136 and the species name.

## Abbreviations

Top5_One-tissue_: genes with an expression level in the top 5% in one tissue type only, and not in the other studied tissues
FPKM: frequency per kilobase million
MWU-test: Mann-Whitney U-test
