# Supplementary material for: Adaptation of codon and amino acid use for translational functions in highly expressed cricket genes
Source: BMC Genomics. 2021 Apr 6;22:234. doi: 10.1186/s12864-021-07411-w (PMC8022432; doi:10.1186/s12864-021-07411-w)
Supplement: Supplementary file 1 — Additional file 1. The file contains the Supplementary Tables, Figures and Text which are denoted and Tables S1 to S6, Figure S1, and Text File S1. [file 12864_2021_7411_MOESM1_ESM.pdf]

ADDITIONAL FILE 1

Adaptation of codon and amino acid use for translational functions in highly expressed cricket genes

Authors: Carrie A. Whittle[1], Arpita Kulkarni[1], Nina Chung[1], Cassandra G. Extavour[1,2]

1. Department of Organismic and Evolutionary Biology, Harvard University, 16 Divinity Avenue, Cambridge MA USA
2. Department of Molecular and Cellular Biology, Harvard University, 16 Divinity Avenue, Cambridge MA USA

Table. S1. The RNA-seq datasets for each of the male and female tissue types under study for *G. bimaculatus*. The number of reads (single-end) before and after trimming with BBduk (https://jgi.doe.gov/data-and-tools/bbtools) is shown. The data are available at the Short Read Archive (SRA) under the project identifier PRJNA564136 (study ID SRP220521, released upon publication). See also [1].

			**No. Reads**	
**Sex**	**Tissues**	**Sample Name**	**Before trimming**	**After trimming**
Male 1	Accessory gland	AK-28_S6.R1	8,519,999	8,455,381
	Brain	AK-25_S3.R1	10,927,264	10,543,501
	Somatic reproductive system	SHC-18_S14.R1	32,497,283	32,430,843
	Testes	SHC-17_S13.R1	19,928,912	19,751,731
	Ventral nerve cord	AK-26_S4.R1	11,488,521	11,140,299
Male 2	Accessory gland	AK-35_S13.R1	15,110,718	14,973,668
	Brain	AK-32_S10.R1	18,039,328	17,850,399
	Somatic reproductive system	AK-31_S9.R1	11,993,680	11,702,596
	Testes	AK-30_S8.R1	13,672,147	13,529,248
	Ventral nerve cord	AK-33_S11.R1	11,677,747	11,445,159
Female 1	Brain	AK-39_S17.R1	13,920,966	13,750,206
	Ovary	AK-37_S15.R1	21,725,208	21,128,416
	Somatic reproductive system	AK-38_S16.R1	13,870,827	13,718,497
	Ventral nerve cord	AK-40_S18.R1	12,599,661	12,341,413
Female 2	Brain	AK-45_S23.R1	19,312,301	19,036,974
	Ovary	AK-43_S21.R1	27,627,122	27,049,583
	Somatic reproductive system	AK-44_S22.R1	11,688,814	11,539,571
	Ventral nerve cord	AK-46_S24.R1	13,591,143	13,143,568

Table S2. The ΔRSCU for each of the nine tissues under study using genes with Top5$_{\text{One-tissue}}$ status per tissue type (versus genes with the lowest 5% expression level per tissue type). *P<0.05,**P<0.001. Note that the nongonadal tissues had fewer genes with Top5$_{\text{One-tissue}}$ expression than those with gonadal expression, particularly for the brain, and thus inherently had lower power of t-tests. However, the largest ΔRSCU per amino acid for each of the nine tissues is underlined and in bold face for all tissues irrespective of shown P value to show the tendency for high congruency among tissues. N values for the Top5$_{\text{One-tissue}}$ genes are as follows: ovary (274), testis (270), female somatic reproductive system (67), male somatic reproductive system (104), female brain (24), male brain (22), female ventral nerve cord (32), male ventral nerve cord (33), and male accessory gland (162).

Amino Acid	Codon	Organism wide	P	Ovary	P	Testis	P	Fem somatic reproductive system	P	Male somatic reproductive system	P	Female brain	P	Male brain	P	Fem ventral nerve cord	P	Male ventral nerve cord	P	Male Acc. Gland	P
Ala	GCT	**+0.871**	**	**+0.879**	**	**+0.914**	**	**+0.723**	**	**+0.249**	*	**+0.483**	*	**+0.663**	*	**+0.339**	*	**+0.376**	**	**+0.773**	**
Ala	GCC	-0.344	**	-0.584	**	-0.650	**	-0.375	**	+0.002		-0.154		-0.299	*	-0.158		-0.182	*	-0.510	**
Ala	GCA	+0.518	**	+0.756	**	+0.836	**	+0.416	**	+0.140		+0.342	**	+0.370	*	+0.147	*	+0.292	**	+0.536	**
Ala	GCG	-1.039	**	-1.041	**	-1.104	**	-0.750	**	-0.378	**	-0.652	**	-0.714	*	-0.304	*	-0.465	**	-0.839	**
Arg	CGT	+0.463	**	+0.387	**	+0.437	**	+0.442	**	+0.041		**+0.490**		+0.111		+0.101		-0.025		+0.236	**
Arg	CGC	-1.053	**	-1.552	**	-1.658	**	-0.801	**	-0.299		-0.537		-0.548		-0.597	**	-0.780	*	-1.295	**
Arg	CGA	+0.185	**	+0.183	*	+0.364	**	+0.137	*	+0.027		+0.431		+0.102		+0.081		+0.343	*	+0.279	*
Arg	CGG	-0.548	**	-0.520	**	-0.575	**	-0.379	**	-0.216	*	-0.349	*	-0.464	*	-0.005		-0.226		-0.453	**
Arg	AGA	**+0.881**	**	**+1.296**	**	**+1.296**	**	**+0.645**	**	**+0.370**	*	+0.190		+0.392		**+0.361**	*	**+0.538**	*	**+1.123**	**
Arg	AGG	+0.047		+0.203	**	+0.159	**	-0.014		+0.105		-0.197		**+0.436**		-0.092		+0.187		+0.057	
Asn	AAT	**+0.416**	**	**+0.661**	**	**+0.713**	**	**+0.340**	*	**+0.086**		**+0.306**		**+0.262**		**+0.226**		**+0.252**		**+0.610**	*
Asn	AAC	-0.244	**	-0.552	**	-0.594	**	-0.213	*	+0.021		-0.176		-0.307		-0.096		-0.297	*	-0.500	
Asp	GAT	**+0.520**	**	**+0.695**	**	**+0.801**	**	**+0.513**	**	**+0.132**		**+0.333**		**+0.156**		**+0.380**	*	**+0.366**	*	**+0.588**	**
Asp	GAC	-0.482	**	-0.669	**	-0.761	**	-0.465	**	-0.161	*	-0.285		-0.199		-0.333	*	-0.374	*	-0.563	**
Cys	TGT	**+0.368**	**	**+0.659**	**	**+0.698**	**	**+0.346**	**	**+0.155**	*	**+0.201**	*	**+0.217**		**+0.390**	*	**+0.182**	*	**+0.504**	**
Cys	TGC	-0.365	**	-0.594	**	-0.552	**	-0.284	**	-0.118		-0.207		-0.245		-0.236	*	-0.142		-0.461	**
Gln	CAA	**+0.254**	**	**+0.496**	**	**+0.535**	**	**+0.276**	**	**+0.057**		**+0.101**		-0.048		**+0.093**		**+0.101**		**+0.404**	**
Gln	CAG	-0.218	**	-0.447	**	-0.492	**	-0.224	**	-0.062		-0.048		**+0.098**		-0.094		-0.226	*	-0.371	*
Glu	GAA	**+0.496**	**	**+0.649**	**	**+0.722**	**	**+0.334**	**	**+0.146**	*	**+0.277**	*	**+0.215**	*	**+0.209**	*	**+0.145**		**+0.550**	**
Glu	GAG	-0.480	**	-0.621	**	-0.695	**	-0.311	**	-0.142	*	-0.337	*	-0.284	*	-0.183	*	-0.238	*	-0.527	**
Gly	GGT	**+0.610**	**	+0.662	**	+0.647	**	**+0.485**	**	+0.152		+0.350	*	**+0.504**	*	**+0.243**	*	+0.176	*	**+0.586**	**
Gly	GGC	-0.709	**	-1.067	**	-1.109	**	-0.606	**	-0.139		-0.657	**	-0.431		-0.374		-0.395	**	-0.864	**
Gly	GGA	+0.483	**	**+0.714**	**	**+0.775**	**	+0.367	**	**+0.311**	*	**+0.437**	*	-0.097		+0.251		**+0.441**	*	+0.573	*
Gly	GGG	-0.383	**	-0.320	**	-0.298	**	-0.229	*	-0.307	**	-0.104		+0.049		-0.091		-0.190		-0.278	

His	CAT	**+0.511**	**	**+0.712**	**	**+0.724**	**	**+0.434**	**	**+0.261**	*	**+0.192**		**+0.205**		**+0.160**		**+0.405**	*	**+0.560**	**
His	CAC	-0.452	**	-0.682	**	-0.646	**	-0.332	**	-0.168		-0.307		-0.342		-0.028		-0.266		-0.568	**
Ile	ATT	**+0.603**	**	**+0.658**	**	**+0.731**	**	**+0.496**	**	**+0.207**		**+0.595**	*	**+0.403**		+0.181		**+0.284**		**+0.587**	**
Ile	ATC	-0.452	**	-0.839	**	-0.944	**	-0.480	**	-0.081		-0.482	*	-0.351		-0.278		-0.191		-0.709	**
Ile	ATA	+0.045		+0.318	**	+0.392	**	+0.062		-0.015		-0.071		-0.024		**+0.265**		-0.100		+0.263	
Leu	TTA	**+0.537**	**	**+0.843**	**	**+0.930**	**	**+0.454**	**	**+0.166**	*	**+0.519**	*	**+0.257**		+0.112		**+0.449**	*	**+0.663**	**
Leu	TTG	+0.383	**	+0.585	**	+0.553	**	+0.324		+0.102		+0.077		+0.059		+0.127		+0.130		+0.560	
Leu	CTT	+0.409	**	+0.524	**	+0.557	**	+0.414	**	+0.041		+0.284		+0.195		**+0.417**	*	+0.192	*	+0.436	**
Leu	CTC	-0.629	**	-0.804	**	-0.778	**	-0.492	**	-0.112		-0.358		-0.304		-0.213		-0.254		-0.625	**
Leu	CTA	+0.007		+0.144	**	+0.159	**	+0.086	*	-0.008		+0.058		+0.066		-0.064		-0.065		+0.145	*
Leu	CTG	-0.692	**	-1.280	**	-1.409	**	-0.778	**	-0.180		-0.576		-0.264		-0.370		-0.628	*	-1.169	**
Lys	AAA	**+0.263**	**	**+0.488**	**	**+0.565**	**	**+0.247**		**+0.059**		**+0.133**		**+0.221**		**+0.184**		**+0.069**		**+0.482**	
Lys	AAG	-0.160	**	-0.421	**	-0.505	**	-0.203	*	+0.015		-0.224		-0.139		-0.173		-0.159		-0.413	*
Phe	TTT	**+0.407**	**	**+0.666**	**	**+0.707**	**	**+0.350**	**	**+0.152**	**	**+0.332**	*	**+0.309**	*	**+0.332**	*	**+0.146**	*	**+0.513**	**
Phe	TTC	-0.265	**	-0.584	**	-0.614	**	-0.277	**	-0.049	*	-0.221	*	-0.203	*	-0.290	*	-0.154		-0.415	**
Pro	CCT	**+0.749**	**	**+0.737**	**	+0.828	**	**+0.788**	**	**+0.279**	*	+0.351		**+0.364**		**+0.452**	**	**+0.418**	*	+0.615	**
Pro	CCC	-0.359	**	-0.600	**	-0.659	**	-0.504	**	-0.019		-0.292		-0.178		-0.289	*	-0.366	*	-0.580	**
Pro	CCA	+0.483	**	+0.732	**	**+0.873**	**	+0.497	**	+0.165		**+0.517**	*	+0.226		+0.266	*	+0.330	*	**+0.683**	**
Pro	CCG	-0.843	**	-0.900	**	-0.998	**	-0.727	**	-0.371	**	-0.521	*	-0.537	*	-0.367	*	-0.562	**	-0.700	**
Ser	TCT	**+0.731**	**	+0.691	**	+0.770	**	+0.379	*	+0.148		+0.102		**+0.530**		+0.141		+0.271		**+0.610**	*
Ser	TCC	-0.208	**	-0.484	**	-0.554	**	-0.326		+0.039		-0.082		-0.452	*	-0.305	*	-0.264		-0.479	
Ser	TCA	+0.493	**	+0.708	**	**+0.855**	**	**+0.568**	**	**+0.350**	*	**+0.498**	*	+0.223		**+0.457**	*	+0.326	*	+0.595	**
Ser	TCG	-0.723	**	-0.843	**	-0.925	**	-0.551	**	-0.406	**	-0.683	**	-0.357		-0.460	**	-0.498	**	-0.696	**
Ser	AGT	+0.325	**	**+0.716**	**	+0.630	**	+0.387	*	+0.058		+0.436	*	+0.327		+0.212		**+0.424**		+0.600	*
Ser	AGC	-0.619	**	-0.773	**	-0.763	**	-0.443	**	-0.176		-0.258		-0.259		-0.026		-0.243		-0.619	**
Thr	ACT	**+0.644**	**	+0.724	**	+0.797	**	+0.452	**	**+0.222**	*	+0.323	*	**+0.510**	*	**+0.447**	**	**+0.324**		**+0.633**	**
Thr	ACC	-0.223	**	-0.487	**	-0.547	**	-0.262	**	+0.050		-0.110		-0.337		-0.213		-0.106		-0.346	*
Thr	ACA	+0.493	**	**+0.783**	**	**+0.868**	**	**+0.469**	**	+0.163	*	**+0.586**	**	+0.150		+0.304	*	+0.205	*	+0.629	**
Thr	ACG	-0.873	**	-0.997	**	-1.077	**	-0.624	**	-0.439	**	-0.758	*	-0.288		-0.501	*	-0.498	**	-0.906	**
Tyr	TAT	**+0.430**	**	**+0.671**	**	**+0.668**	**	**+0.442**	**	**+0.203**	*	**+0.214**		**+0.295**		**+0.302**	*	**+0.306**		**+0.554**	**
Tyr	TAC	-0.186	**	-0.466	**	-0.477	**	-0.229	*	-0.009		-0.156		-0.064		-0.268	*	-0.181		-0.441	*
Val	GTT	**+0.600**	**	**+0.787**	**	**+0.788**	**	**+0.474**	**	+0.104		+0.252	*	**+0.313**	*	+0.073		**+0.288**		**+0.709**	**
Val	GTC	-0.394	**	-0.474	**	-0.535	**	-0.361	**	-0.037		-0.136		+0.040		-0.336	*	-0.199		-0.377	**
Val	GTA	+0.314	**	+0.435	**	+0.493	**	+0.247	**	**+0.112**	*	**+0.302**	*	+0.255		**+0.196**	*	+0.138	*	+0.347	**

Val	GTG	-0.484	**	-0.725	**	-0.741	**	-0.340	**	-0.197	*	-0.397	*	-0.587	-0.039	-0.329	*	-0.661	**

Table S3. Top predicted GO functional groups for organism-wide highly expressed genes (top 5% expression levels when averaged FPKM across all nine tissues) with elevated use (RSCU≥1.5) of wobble codons. Results are also shown with elevated use of the same wobble codons for genes with the top 5% expression within the ovaries and testes and not in any other tissues (Top5$_{One-tissue}$). The clusters with the greatest enrichment (abundance) scores are shown per category. P-values are derived from a modified Fisher's test, where lower values indicate greater enrichment. Data is from DAVID software [2] using those *G. bimaculatus* genes with *D. melanogaster* orthologs (BLASTX [3]).

			**Organism wide**		
**GGT Gly**			**GAT Asp**		
Cluster 1	Enrichment Score: 11.12	P value	Cluster 1	Enrichment Score: 8.77	P value
	Cytoplasmic translation	2.20X10^{-21}		Cytoplasmic translation	3.80X10^{-16}
	Ribosomal protein	4.10X10^{-18}		Ribosomal protein	1.70X10^{-14}
Cluster 2	Enrichment Score: 8.77		Cluster 2	Enrichment Score: 5.56	
	Mitochondrion inner membrane	4.20X10^{-11}		Mitochondrion	1.20X10^{-9}
	Mitochondrion	4.80X10^{-10}		Mitochondrion inner membrane	2.00X10^{-5}
Cluster 3	Enrichment Score: 5.36		Cluster 3	Enrichment Score: 4.89	
	Mitochondrion	4.80X10^{-10}		Mitochondrion	1.20X10^{-9}
	Transit peptide	1.50X10^{-5}		Transit peptide	2.70X10^{-4}
**CAT His**			**TAT Tyr**		
Cluster 1	Enrichment Score: 9.8	P value	Cluster 1	Enrichment Score: 5.78	P value
	Cytoplasmic translation	1.60X10^{-19}		Mitochondrion inner membrane	6.80X10^{-12}
	Ribosomal protein	1.70X10-^{16}		Oxidative phosphorylation	6.40X10^{-8}
Cluster 2	Enrichment Score: 8.61		Cluster 2	Enrichment Score: 3.24	
	Mitochondrion	3.10X10^{-11}		Electron transport	9.90X10^{-5}
	Mitochondrion inner membrane	1.00X10^{-9}		Respiratory chain	9.20X10^{-4}
Cluster 3	Enrichment Score: 6.72		Cluster 3	Enrichment Score: 2.98	
	Oxidative phosphorylation	6.40X10^{-11}		Cytoplasmic translation	1.50X10^{-7}
	Oxidoreductase	4.00X10^{-10}		Ribosome	4.50X10^{-5}
			**Top5$_{One-tissue}$ Ovaries**		
**GGT Gly**			**GAT Asp**		
Cluster 1	Enrichment Score: 1.93	P value	Cluster 1	Enrichment Score: 2.35	P value
	Helicase	3.50X10^{-4}		Eggshell chorion gene amplification	1.30X10^{-5}
	DNA/RNA helicase, DEAD/DEAH box type	3.50X10^{-3}		Cell cycle	5.80X10^{-2}
	P-loop nucleoside triphosphate hydrolase	1.10X10^{-2}		Cell division	1.20X10^{-1}
	ATP-binding	5.50X10^{-2}	Cluster 2	Enrichment Score: 1.78	
Cluster 2	Enrichment Score: 1.38			Eggshell chorion gene amplification	1.30X10^{-5}
	Nuclear pore	1.10X10^{-2}		DNA binding	4.00X10^{-1}
	Protein transporter activity	2.70X10^{-2}	Cluster 3	Enrichment Score: 1.43	
Cluster 3	Enrichment Score: 1.1			Protein transport	2.90X10^{-3}
	Nucleus	8.20X10^{-3}		Neurotransmitter secretion	2.50X10^{-2}
**CAT His**			**TAT Tyr**		
Cluster 1	Enrichment Score: 1.45	P value	Cluster 1	Enrichment Score: 1.99	P value
	Zinc	1.00X10^{-2}		RNA secondary structure unwinding	2.80X10^{-4}
	Metal-binding	5.40X10^{-2}		RNA helicase, DEAD-box type, Q motif	5.00X10^{-4}
Cluster 2	Enrichment Score: 1.2			ATP-dependent RNA helicase activity	1.30X10^{-3}
	Protein transport	1.90X10^{-2}		NucleotidX10-binding	2.60X10^{-2}
	Transport	1.10X10^{-1}		Hydrolase	3.40X10^{-1}
Cluster 3	Enrichment Score: 1.04		Cluster 2	Enrichment Score: 1.23	
	Ubiquitin-protein transferase activity	1.40X10^{-2}		WD40	3.20X10^{-2}
	Zinc-finger	2.00X10^{-2}		WD40/YVTN repeat-likX10-containing domain	8.80X10^{-2}
	Protein polyubiquitination	8.50X10^{-2}	Cluster 3	Enrichment Score: 1.16	
	Zinc finger, RING/FYVE/PHD-type	2.70X10^{-1}		ATP-binding	8.20X10^{-3}
	Zinc ion binding	5.90X10^{-1}		NucleotidX10-binding	2.60X10^{-2}

Top5$_{\text{One-tissue}}$ Testes

GGT Gly

Cluster 1	Enrichment Score: 1.72	P value
	Protein import into nucleus	4.80X10^{-3}
	Armadillo-type fold	8.90X10^{-3}
	Armadillo-like helical	1.20X10^{-2}
	Protein transporter activity	1.30X10^{-2}
	Cytosol	3.70X10^{-1}
Cluster 2	Enrichment Score: 0.91	
	Mitochondrion inner membrane	4.00X10^{-2}
	Transmembrane region	3.20X10^{-1}
Cluster 3	Enrichment Score: 0.67	
	Transmembrane helix	1.90X10^{-1}
	Membrane	2.00X10^{-1}

GAT Asp

Cluster 1	Enrichment Score: 2.26	P value
	Ubl conjugation pathway	1.50X10^{-3}
	Thiol-dependent ubiquitin-specific protease activity	3.30X10^{-3}
	Protein deubiquitination	3.40X10^{-3}
	Protease	5.60X10^{-2}
Cluster 2	Enrichment Score: 1.56	
	Zinc finger, RING/FYVE/PHD-type	8.80X10^{-3}
	Metal-binding	1.90X10^{-2}
Cluster 3	Enrichment Score: 0.88	
	Mitosis	8.20X10^{-2}
	Cell cycle	2.50X10^{-1}

CAT His

Cluster 1	Enrichment Score: 2	P value
	Dual specificity phosphatase	2.40X10^{-3}
	Protein tyrosine/serine/threonine phosphatase	6.50X10^{-3}
	Protein dephosphorylation	1.20X10^{-1}
Cluster 2	Enrichment Score: 1.68	
	Zinc	3.10X10^{-3}
	Metal-binding	1.70X10^{-2}
	Ubl conjugation pathway	2.90X10^{-2}
Cluster 3	Enrichment Score: 1.57	
	Zinc ion binding	1.10X10^{-2}
	Zinc finger, RING/FYVE/PHD-type	1.40X10^{-2}
	Ubiquitin-protein transferase activity	3.30X10^{-2}
	Protein ubiquitination	5.70X10^{-2}

TAT Tyr

Cluster 1	Enrichment Score: 1.63	P value
	Cell cycle	1.20X10^{-2}
	Mitosis	2.30X10^{-2}
Cluster 2	Enrichment Score: 0.95	
	G-protein beta WD-40 repeat	2.40X10^{-2}
	WD40/YVTN repeat-likX10-containing domain	2.30X10^{-1}
Cluster 3	Enrichment Score: 0.88	
	ZnF_C2H2	8.20X10^{-2}
	Zinc finger C2H2-type/integrase DNA-binding domain	2.50X10^{-1}

Table S4. The size/complexity scores, hydropathy, and protein folding characteristics for each of the 20 amino acids. These data were used for analysis of amino acid usage [4-6].

Amino acid	S/C score	Hydrophobic score	Folding property
Gly	1	-0.4	breaker
Ala	4.76	1.8	alpha
Val	12.28	4.2	beta
Ile	16.04	4.5	beta
Leu	16.04	3.8	alpha
Ser	17.86	-0.8	breaker
Thr	21.62	-0.7	beta
Lys	30.14	-3.9	alpha
Pro	31.8	-1.6	breaker
Asp	32.72	-3.5	breaker
Asn	33.72	-3.5	breaker
Glu	36.48	-3.5	alpha
Gln	37.48	-3.5	alpha
Phe	44	2.8	alpha
Arg	56.34	-4.5	alpha
Tyr	57	-1.3	beta
Cys	57.16	2.5	beta
His	58.7	-3.2	alpha
Met	64.68	1.9	alpha
Trp	73	-0.9	beta

Table S5. The average amino acid use of the $Top5_{One\text{-}tissue}$ genes in *G. bimaculatus* (frequency) for each of nine tissue types. Genes had to be in the top 5% expession iin only one tissue type and not in the top 5% of other tissues. Differences between male- and female-paired tissues are shown. **Indicates P<0.05 using a t-test between males and females for each tissue, *indicates P<0.1 and thus is a putative difference. Values for male accessory glands are also shown. The percent differences (Diff.) is indicated for females versus males (female demoninator). The largest three statistically signficant values per tissue are in bold.

		**Gonad**				**Somatic reproductive system**				**Brain**				**Ventral nerve cord**				**Accessory glands**
		Frequency				Frequency				Frequency				Frequency				Frequency
Amino acid	S/C score	Female	Male	Diff.	P	Female	Male	Diff.	P	Female	Male	Diff.	P	Female	Male	Diff.	P	Male
Gly	1	5.25	5.55	-5.69	*	6.29	6.89	-9.54		5.79	7.62	**-31.57**	*	6.01	6.91	-14.99		6.75
Ala	4.76	6.20	6.29	-1.45		7.31	8.46	**-15.75**	*	7.85	8.68	-10.51		9.30	8.33	10.45		6.91
Val	12.28	6.84	6.45	**5.70**	**	6.59	6.61	-0.25		6.66	5.65	15.07		6.83	6.94	-1.62		6.80
Ile	16.04	5.43	5.47	-0.66		5.01	4.67	6.73	*	5.14	5.24	-1.91	**	4.61	4.66	-0.95		4.23
Leu	16.04	9.61	9.37	2.46		9.18	8.29	9.70		10.12	8.74	13.61		9.33	8.39	10.05		9.00
Ser	17.86	7.94	7.73	2.68		7.23	7.21	0.25		7.31	8.63	-18.09		7.96	6.89	13.40		7.47
Thr	21.62	5.09	5.13	-0.84		5.70	4.94	13.42		5.58	4.49	19.46	**	5.26	5.29	-0.75		5.01
Lys	30.14	6.56	6.73	-2.61		5.72	5.68	0.70		4.91	5.56	-13.19		5.29	5.43	-2.71		4.95
Pro	31.8	4.51	4.71	-4.43		4.74	6.07	**-27.88**	**	5.35	4.68	12.56		5.65	6.04	-7.00		5.68
Asp	32.72	5.30	5.27	0.65		5.83	4.95	**15.14**	**	4.77	4.24	11.18		4.96	4.39	11.50		4.70
Asn	33.72	4.55	4.65	-2.22		4.35	4.06	6.71	*	4.03	3.95	1.86		4.11	3.64	11.45		3.62
Glu	36.48	6.82	7.32	**-7.32**	**	6.84	6.20	9.45		6.04	4.97	17.77		5.97	6.05	-1.25		6.36
Gln	37.48	4.03	4.12	-2.28		4.12	3.72	9.85		3.81	3.49	8.50		4.27	3.66	14.32		4.05
Phe	44	4.13	4.09	0.89		3.54	3.86	-9.05		4.63	4.77	-3.18	**	3.81	3.37	11.51		3.56
Arg	56.34	5.48	5.13	**6.33**	*	5.83	6.22	-6.64	**	5.51	6.68	**-21.15**	**	6.80	7.15	-5.09		7.51
Tyr	57	3.09	2.96	4.10		2.88	3.29	-14.02		3.82	3.00	**21.54**	**	2.62	2.91	-11.06		2.65
Cys	57.16	2.44	2.31	5.12		2.33	2.51	-7.77		1.98	2.72	-37.65		2.31	3.26	-41.20		2.44
His	58.7	2.53	2.69	-6.03		2.44	2.27	6.89		2.13	2.70	-27.18		2.63	2.25	**14.51**	*	2.53
Met	64.68	2.58	2.55	1.29		2.48	2.38	3.91		2.68	2.60	3.06		2.28	2.70	**-18.63**	*	2.37
Trp	73	1.23	1.16	6.00		1.24	1.25	-0.77		1.50	0.94	36.94		1.34	1.15	13.74		1.28

Table S6. The Spearman Ranked correlations of the average amino acid frequency for genes with Top5$_{\text{One-tissue}}$ status in *G. bimaculatus*. Data were used from Table S5 to determine correlations across all 20 amino acids between pairs of female and pairs of male tissues. ** Indicates P<0.001.

**Female tissues (R-values)**							**Male tissues (R-values)**								
**Female tissues**	Gonad	P	SRS	P	Brain	P	**Male tissues**	Gonad	P	SRS	P	Brain	P	VNC	P
Somatic reproductive system	0.948	**					Somatic reproductive system	0.894	**						
Brain	0.907	**	0.958	**			Brain	0.884	**	0.95	**				
Ventral nerve cord	0.904	**	0.957	**	0.956	**	Ventral nerve cord	0.861	**	0.973	**	0.938	**		
							Male Accessory glands	0.87	**	0.964	**	0.925	**	0.977	**

Notes: SRS=somatic reproductive system, VNC=ventral nerve cord.

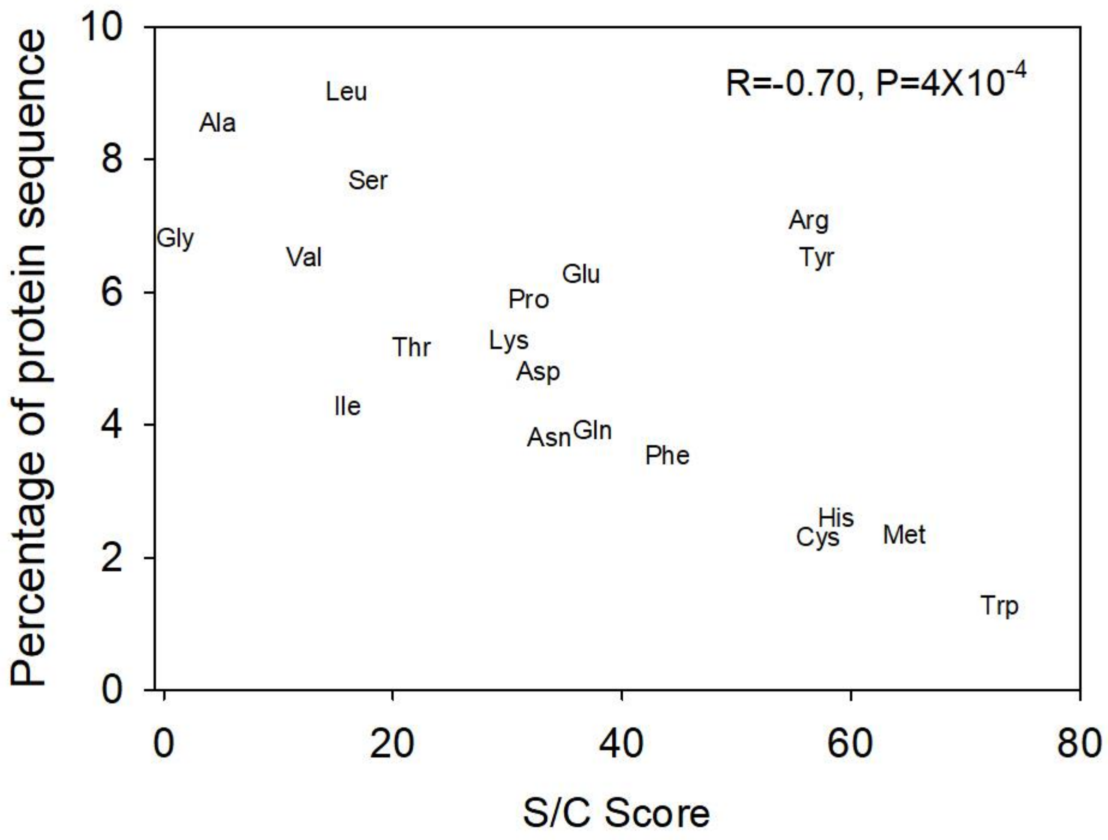

Fig. S1. The relationship between amino acid usage (percent per gene, averaged across all genes) and size/complexity (S/C) score across all 15,539 annotated genes in *G. bimaculatus*. Spearman's R and P values are shown.

Text File S1

Biased Gene Conversion

A possible factor that could contribute to AT3 content of genes is biased gene conversion (BGC) [7, 8]. For instance, it has been reported that errors during DNA repair can lead to enhanced GC content of genes, due to favoring of GC insertions in mismatch repair of strand breaks resulting from meiotic recombination, that can ultimately enhance GC content (and thus lack of BGC may enhance AT content) [9-11]. In humans, BGC conversion was found to be more common in lowly than highly expressed genes in the germ cells during meiosis, which was interpreted as reflecting greater crossing-over events in genes exhibiting low expression (or, inhibition of crossing-over events in genes with high meiotic expression) [8, 12]. Codon use in humans, which have large GC-rich isochores in the genome [8], may be more apt to be affected by a relationship between expression during meiosis and intragenic recombination rates (where recombination is controlled by PRMD9 and directly affects CDS) [8] than organisms with different recombination mechanisms (e.g., concentrated to promotors) and landscapes such as other vertebrates [13] or some insects [14], and possibly including the crickets studied here. We nonetheless considered this hypothesis in our present study. If we assume there may be parallels to human patterns in these insects, then we can consider this hypothesis using the results for the $Top5_{One\text{-}tissue}$ datasets, where optimal codons were determined for each tissue type using only genes that were in top 5% expressed genes in one tissue, and not in the top 5% in any other tissue (could be expressed at any lower level in other tissues). By definition, these nine $Top5_{One\text{-}tissue}$ gene datasets are thus all mutually exclusive of each other (Table S2). Under the hypothesis described above [8], the high AT3 (or low GC3) of highly expressed ovary and testis genes ($Top5_{One\text{-}tissue}$) observed here in *G. bimaculatus* in Table S2 could possibly result from lower BGC of genes (reduced crossing over) of genes highly transcribed in the male and in the female gonads containing the meiotic cells (than lowly expressed genes). Significantly, however, we found highly similar A3 or T3 optimal codons in each of the nine distinct tissue types, including those highly expressed in the male and female meiotic tissues (that is, those with $Top5_{One\text{-}tissue}$ status in the testis, and those from the ovary), and for all other seven studied tissues wherein meiosis does not occur (male and female somatic reproductive system, brain, and ventral nerve cord, and the male accessory glands, Table S2). This pattern concurs with a model that suggests factors other than BGC in meiosis (or lack thereof in highly expressed meiotic genes) contribute to the enhanced AT3 of the most highly expressed cricket genes (Table 1, Table S2).

In this regard, while this analysis is preliminary and recombination rate data will be essential to further test whether there are relationships between meiotic expression, recombination, and BGC in crickets, the patterns are consistent with our findings suggesting that selection has at least partly contributed to the elevated use of AT3 optimal codons in the most highly expressed genes in this organism. This includes the findings that small increments in AT-I content (of 0.1), which controls for background mutational and BGC pressures [15, 16], yielded marked differences in AT3 of highly and lowly expressed genes (Fig. 1), and that optimal codon use was correlated to tRNA gene counts for a majority of optimal codons (Table 1).

References

1. Whittle CA, Kulkarni A, Extavour CG: **Sex-biased genes expressed in the cricket brain evolve rapidly**. *BioRxiv* 2020, www.biorxiv.org/content/10.1101/2020.07.07.192039v1
2. Huang da W, Sherman BT, Lempicki RA: **Systematic and integrative analysis of large gene lists using DAVID bioinformatics resources**. *Nat Protoc* 2009, **4**(1):44-57.
3. Altschul SF, Madden TL, Schaffer AA, Zhang J, Zhang Z, Miller W, Lipman DJ: **Gapped BLAST and PSI-BLAST: a new generation of protein database search programs**. *Nucleic Acids Research* 1997, **25**(17):3389-3402.
4. Sabbia V, Piovani R, Naya H, Rodriguez-Maseda H, Romero H, Musto H: **Trends of amino acid usage in the proteins from the human genome**. *Journal of Biomolecular Structure and Dynamics* 2007, **25**(1):55-59.
5. Kyte J, Doolittle RF: **A simple method for displaying the hydropathic character of a protein**. *Journal of Molecular Biology* 1982, **157**(1):105-132.
6. Dufton MJ: **Genetic code synonym quotas and amino acid complexity: cutting the cost of proteins?** *Journal of Theoretical Biology* 1997, **187**(2):165-173.
7. Galtier N, Roux C, Rousselle M, Romiguier J, Figuet E, Glemin S, Bierne N, Duret L: **Codon Usage Bias in Animals: Disentangling the Effects of Natural Selection, Effective Population Size, and GC-Biased Gene Conversion**. *Molecular Biology and Evolution* 2018, **35**(5):1092-1103.
8. Pouyet F, Mouchiroud D, Duret L, Semon M: **Recombination, meiotic expression and human codon usage**. *Elife* 2017, **6**.

9. de Proce SM, Zeng K, Betancourt AJ, Charlesworth B: **Selection on codon usage and base composition in *Drosophila americana***. *Biology Letters* 2012, **8**(1):82-85.
10. Zeng K, Charlesworth B: **Studying patterns of recent evolution at synonymous sites and intronic sites in *Drosophila melanogaster***. *Journal of Molecular Evolution* 2010, **70**(1):116-128.
11. Marais G: **Biased gene conversion: implications for genome and sex evolution**. *Trends in Genetics* 2003, **19**(6):330-338.
12. McVicker G, Green P: **Genomic signatures of germline gene expression**. *Genome Research* 2010, **20**(11):1503-1511.
13. Baker Z, Schumer M, Haba Y, Bashkirova L, Holland C, Rosenthal GG, Przeworski M: **Repeated losses of PRDM9-directed recombination despite the conservation of PRDM9 across vertebrates**. *Elife* 2017, **6**.
14. Smukowski Heil CS, Ellison C, Dubin M, Noor MA: **Recombining without Hotspots: A Comprehensive Evolutionary Portrait of Recombination in Two Closely Related Species of Drosophila**. *Genome Biol Evol* 2015, **7**(10):2829-2842.
15. Chamary JV, Hurst LD: **Similar rates but different modes of sequence evolution in introns and at exonic silent sites in rodents: evidence for selectively driven codon usage**. *Molecular Biology and Evolution* 2004, **21**(6):1014-1023.
16. Ingvarsson PK: **Gene expression and protein length influence codon usage and rates of sequence evolution in Populus tremula**. *Molecular Biology and Evolution* 2007, **24**(3):836-844.
